# Professional development program to promote students’ conceptual understanding through technology-enhanced teaching: a learner-centered evaluation

**DOI:** 10.3389/fpsyg.2025.1666808

**Published:** 2025-10-03

**Authors:** Susanne Digel, Jürgen Roth

**Affiliations:** Department of Natural and Environmental Sciences, Mathematics Education for Secondary Schools, Institute for Mathematics, RPTU University of Kaiserslautern-Landau, Landau, Germany

**Keywords:** technology-enhanced teaching, teacher professional development program, TPDP impact, TPDP transfer process, conceptual understanding, basic mental models

## Abstract

**Introduction:**

The digital transformation of schools is currently in progress; yet, the beneficial utilization of technology-enhanced teaching (TET) for students’ learning remains an intricate endeavor. Teachers report a lack of skills in integrating technology to support learning and call for teacher professional development programs (TPDPs) and best practice materials. This paper outlines the concept and evaluation design of the TPDP MaTeGnu for upper secondary schools. MaTeGnu aims to exploit the potential of technology for instructional quality to support students’ conceptual learning with a focus on basic mental models. Based on a research synthesis on influential factors of TPDPs we formulate a TPDP model to facilitate the crucial transfer process from TPDP offer to teaching and thereof discuss the alignment of the MaTeGnu design with the model.

**Methods:**

The present study aims to evaluate the MaTeGnu TPDP concept by assessing the students’ understanding, as central objective of the project. This evaluation employs an experimental-vs.-control-group design, which involves a comparison of the utilization of basic mental models (BMM) and the conceptual understanding of students regarding the concept of derivatives in classes, where teachers participate in MaTeGnu TPDP (*N_EG_* = 151) and in other classes at the respective schools (*N_CG_* = 571).

**Results:**

Students from MaTeGnu teacher educator classes demonstrate significantly higher conceptual understanding [*t*(225) = 3.78, *p* < 0.001, *d* = 0.346] and utilization of basic mental models, particularly of local rate of change [*t*(267) = 5.17, *p* < 0.001, *d* = 0.474], compared to other students.

**Discussion:**

The findings at the most distant impact level of TPDP reveal noteworthy empirical evidence of the efficacy of the MaTeGnu approach of TET with BMM, particularly the accompanying transfer support, as outlined in our proposed TPDP transfer process model. The emphasis on BMM could provide an effective strategy to implement TET beneficial for learning, even in the more formal setting of upper secondary school mathematics.

## 1 Introduction

Over the past decade, the mathematical achievement of upper secondary school students has become a focal point in educational research, university teaching, and educational policy in Germany (Rolfes et al., 2021). A systematic review of school performance studies in upper secondary schools reveals that the majority of students have significant deficiencies in mathematics competence. More than half of the graduates do not even demonstrate basic understanding of pre-university mathematics. In the light of these findings, the focus on formalism in upper secondary mathematics must be questioned. Although research has elaborated the benefits of technology-enhanced teaching on mathematical understanding (Hillmayr et al., 2020), technology is still rarely used in German classrooms (Fraillon et al., 2020) and teachers often do not fully exploit the potential of technology but use it as substitute in traditional teaching processes (Backfisch et al., 2021). Given teachers’ profound impact on lesson design, it stands to reason that lesson development efficacy depends on their active involvement. Thus, implementing *teacher professional development programs* (TPDPs) has emerged as a pivotal catalyst for advancement within educational institutions. Nevertheless, TPDPs must meet certain conditions to be effective (Lipowsky and Rzejak, 2015, 2019). Unfortunately, there is a paucity of TPDPs that meet even fundamental aspects of these requirements. The TPDP training program, entitled “MaTeGnu” (an acronym for “teaching mathematics with technology sustainably by fostering basic mental models”), has established an ambitious objective: to fulfill as many of the requirements as possible and integrate systematically as well as structurally all relevant institutions and groups involved in teacher education and professional development in the Rhineland-Palatinate region. This approach is designed to ensure sustainable teaching development on a comprehensive scale.

This article introduces the transfer-oriented concept of the MaTeGnu TPDP and reports findings from an evaluation study targeting the most distant level of TPDP impact: student learning (Kirkpatrick and Kirkpatrick, 2016). This level is seldom examined due to the numerous influential factors, which only allows for minor effects. However, it is precisely this level that forms the starting point of the MaTeGnu TPDP, namely the deficient mathematics competences of German upper secondary school students.

## 2 Mathematical understanding of upper secondary school students in Germany: status quo and necessary further development

### 2.1 Status quo

**Figure 1:**
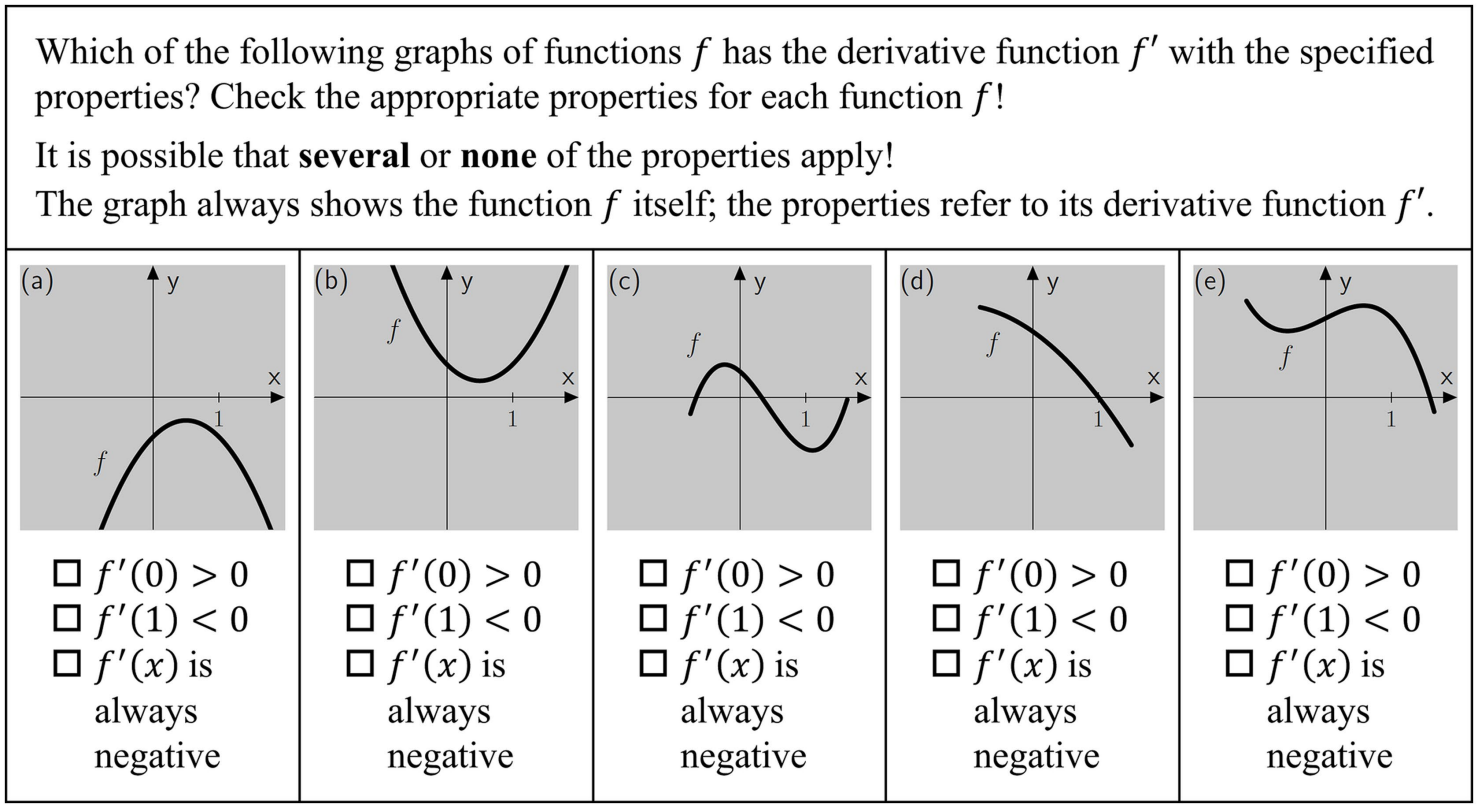
Item Z8PC was translated from Klinger (n.d.), https://www.falke-test.de/, CC BY-NC-SA 4.0.

Rolfes et al. (2021) present an inventory of the findings of school performance studies with survey dates from 1995 onwards, that recorded the mathematics performance of upper secondary school students in Germany. The investigation revealed that the majority of students exhibited evident deficiencies in scientific propaedeutics. Specifically, their capabilities were found to be limited in their ability to engage with learning content typically encountered at the upper secondary level, such as analysis and analytical geometry, among others. The primary objective of the tests utilized in the school performance studies was to evaluate conceptual understanding rather than the application of procedural skills. The activation and utilization of basic mental models (see Chapter 2.3) are imperative for the successful completion of numerous tasks. To solve the sample task illustrated in Figure 1, it is necessary to employ the basic mental model derivative as tangent slope (see Chapter 2.4) (Greefrath et al., 2016).

The secondary analysis by Rolfes et al. (2021) demonstrated that, without exception, in all of the school performance studies examining mathematics performance in upper secondary schools in Germany since 1995, a significant proportion of the student body did not achieve the educational goals of the upper secondary school level in pre-university mathematics, scientific propaedeutics, and study skills.

### 2.2 Necessary further development

One potential explanation for these findings is the predominance of formalisms in upper secondary school mathematics teaching, which frequently remains beyond the grasp of students. This approach is commonly rationalized as a preparatory phase for advanced levels of abstraction that are typically encountered in university-level education. However, as demonstrated by Rach and Ufer (2020), procedural knowledge without a conceptual understanding is insufficient for academic success, while formal representations are dispensable. Conceptual understanding is rooted in the development of basic mental models that guide teaching and learning (Greefrath et al., 2023). The subsequent evolution of upper secondary school pedagogy ought to prioritize fostering conceptual understanding and systematically cultivating basic mental models among students.

### 2.3 What are basic mental models?

The concept of basic mental models (in German, “Grundvorstellungen”) has been firmly established in the field of German-speaking mathematics education for a considerable period. From a didactical perspective, it is employed to delineate the content-related meaning that learners should ascribe to or genuinely confer upon a mathematical concept (vom Hofe and Blum, 2016; Greefrath et al., 2016, 2023).

Basic mental models are instrumental in representing abstract concepts in a clear and coherent manner, thereby facilitating connections between mathematics and real-life situations. Two fundamental categories of basic mental models can be distinguished:

- *Primary basic mental models* are predicated on concrete experiences derived from lessons or everyday life. For instance, children can acquire fundamental cognitive frameworks for the basic mental model correspondence of functions as early as kindergarten, as evidenced by their ability to correctly hang their jackets on coat rack hooks that are labeled with their pictures.
- *Secondary basic mental models* are represented by mathematical means of representation (e.g., number line, coordinate system, graph, term, etc.), especially in more complex mathematical contexts. These basic mental models are acquired and deepened through mental or real operations with them. A secondary basic mental model of the rate of change of a linear function can be acquired and mentally “stored” using a slope triangle on the function graph.

The application of basic mental models to reality is achieved through two mechanisms. Firstly, the recognition of the corresponding mathematical structure in factual contexts is paramount. Secondly, the utilization of these principles in modeling is essential.

In the realm of basic mental models, a dichotomy emerges between individual and normative basic mental models. The former pertains to the cognitive structures developed by students, while the latter is shaped by subject-specific didactic discourse, serving as a foundational framework for the conceptual underpinnings of mathematics. The design of effective tasks and learning environments is intended to facilitate the development of individual basic mental models that are consistent with established normative basic mental models. Accordingly, normative basic mental models function as reference points for pedagogical approaches that prioritize conceptual understanding.

### 2.4 Basic mental models for the concept of derivative

In order to illustrate basic mental models that occur in upper secondary school teaching, we present the basic mental models for the derivative. The concept of *derivative* can be characterized in terms of four basic mental models: (1) *local rate of change* (RC), (2) *tangent slope* (TS), (3) *local linearity* (LL), and (4) *amplification factor* (AF) (Greefrath et al., 2016, 2023).

**Figure 2:**
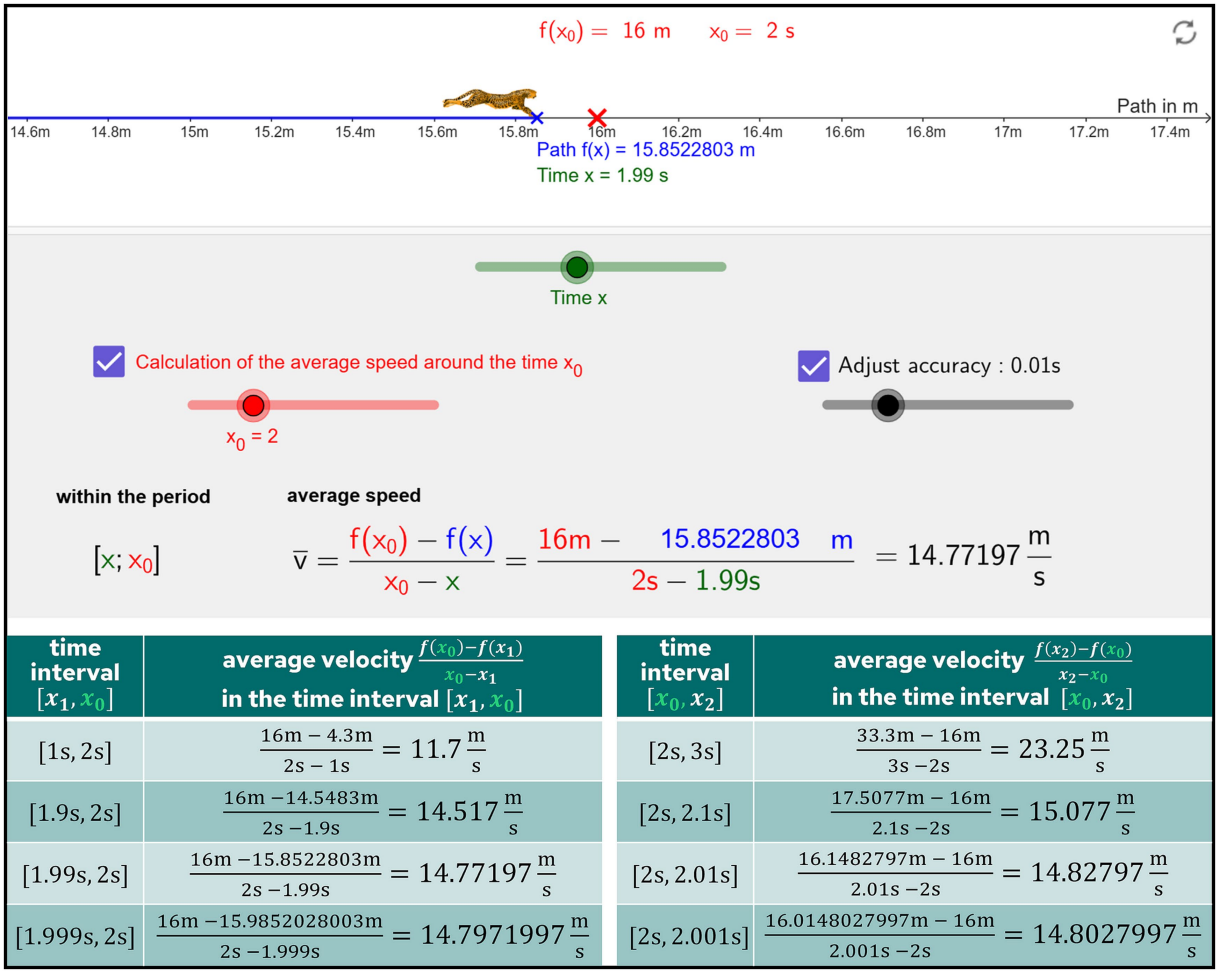
The mean velocity approaches the instantaneous velocity, which is defined as the local rate of change. The requisite data is derived from a GeoGebra-based simulation (https://www.geogebra.org/m/qvjjzxct).

1. The basic mental model of *derivative as local rate of change (RC)* can be worked out using the example of a running cheetah for which the speed at a certain point in time is to be determined. The current location of the cheetah can be determined at any time using suitable measuring devices (e.g., from a GeoGebra-based simulation, see Figure 2). The average velocity in the time interval [2s, 3s] is calculated with the difference quotient $\frac{f(3s)-f(2s)}{3s-2s}=\frac{33.3m-16m}{3s-2s}=23.25\frac{m}{s}$ . If you want to know the current speed at a certain time (e.g., $x_{0}=2s$ ), you can reduce the time interval in which you examine the average speed further and further (Figure 2). The smaller the interval [*x*, *x*_0_], i.e., the closer *x* approaches $x_{0}=2s$ , the closer the mean velocity comes $14.8\frac{m}{s}$ the instantaneous velocity. It comes arbitrarily close. The value that is approximated is called the local rate of change. With this procedure, it is possible to intuitively understand the limiting process from difference quotient to differential quotient $\lim_{x\to x_{0}}\frac{f(x)-f(x_{0})}{x-x_{0}}$ .
2. The basic mental model *derivative as tangent slope (TS)* approaches the derivative geometrically. Learners are acquainted with the concept of the tangent from lower secondary mathematics as a straight line that touches a circle at precisely one point. This conception necessitates refinement towards the analytical perspective of the tangent as a local line of best fit. Otherwise, misunderstandings in the sense of “one-point contact” are inevitable (cf. Greefrath et al., 2023). The conventional approach to the tangent on the function graph is typically motivated geometrically, with the aid of secants, whose slope can be readily ascertained using the coordinates of the two intersection points and the use of slope triangles. The second intersection point can be “dynamized” (e.g., with GeoGebra) to approach the tangential position and observe the difference quotients. The dynamic approach visualizes the gap in the definition of the difference quotient $\frac{f(x)-f(x_{0})}{x-x_{0}}$ at the point $x=x_{0}$ and provides students with an intuitive, visual understanding of the limiting process $\lim_{x\to x_{0}}\frac{f(x)-f(x_{0})}{x-x_{0}}$ .
3. The basic mental model *local linearity (LL)* approaches the derivative from the following vantage point: By gradually focusing on the graph of a differentiable function at a specific point, it becomes evident that the graph approaches a straight line. The derivative of this function is defined as the slope of the line. The utilization of software designed for dynamic mathematics is particularly well-suited for the development of this basic mental model. The process of zooming entails the gradual convergence of the section of the graph displayed on the screen onto a straight line. The basic mental model of local linearity serves as the foundation for various applications. In the context of growth models, the temporal dynamics of population size are frequently characterized in a local manner, that is, within a limited time span, by a linear relationship (Greefrath et al., 2023).
4. The basic mental model *amplification factor (AF)* posits that the derivative functions as a proportionality factor of a functional relationship, thereby signifying the impact of minor variations in the independent variable on the dependent variable. This basic mental model has only recently been acknowledged in mathematics education literature and plays hardly any role in mathematics teaching (Greefrath et al., 2023). One reason for this is that, in contrast to the other three aforementioned models, this basic mental model is not sufficient for developing a fundamental understanding of derivatives. Rather, it is more beneficial as a supplementary tool for specific applications, such as in error calculation.

## 3 Technology-enhanced teaching to support students’ conceptual development

### 3.1 Meta-analysis of studies on technology-enhanced teaching

Technology-enhanced teaching offers the opportunity to provide support for the development of fundamental mathematical understanding. This conclusion is supported by a comprehensive meta-analysis based on a systematic review of studies published since 2000 (*N* = 92), conducted by Hillmayr et al. (2020). The investigation sought to ascertain the potential of technological integration to augment learning in secondary school mathematics and science (grades 5–13). The studies compared the learning outcomes of students who used digital tools with those of a control group that was not taught with digital tools. The meta-analysis revealed that the use of digital tools had a positive effect on student learning outcomes (*g* = 0.65, *p* < 0.001). It is imperative to underscore that technology itself does not inherently engender these effects. The efficacy of these interventions is contingent upon the specific technology employed and the manner in which it is integrated into the educational curriculum. Digel and Roth (2022) contrast in their pre-post-test intervention study two learning environments on functional relationships with a combination of hands-on and digital experiments. One setting (CR) followed the traditional introduction to functional relationships, emphasizing the *correspondence* of an element of the definition set to exactly one element of the set of values. A second setting (CV) prioritized the central aspect of *covariation* of the dependent variable when the independent variable is varied. The covariation setting showed significantly higher learning gains than the correspondence setting (CR: *d* = 0.25, *p* < 0.001; CV: *d* = 0.51, *p* = 0.001).

### 3.2 Summary and necessary next steps

Findings from mathematics education research clearly indicate that using digital learning environments contributes to developing a conceptual understanding and basic mental models help to elicit integration of technology beneficial for understanding. However, schools have not exploited these findings so far. Teachers have reported an absence of consensus on the principles of technology-enhanced teaching that facilitate learning. They have noted a lack of teaching materials elaborated for teaching and a high demand for professional development programs (Mußmann et al., 2021). Thus, the paramount issue is to transform the substantiated research findings into teaching. The ICILS 2023 findings suggest that a crucial strategy for fostering technology-enhanced teaching methods is implementing effective TPDPs that focus on integrating technology in the classroom to enhance conceptual understanding (Fraillon et al., 2025).

## 4 TPDP: a pivotal mechanism for the advancement of teaching processes

Teachers can function as conduits for the implementation of research findings in teaching practice. The initial and secondary phases of teacher education establish the foundation, while the tertiary phase, designated as TPDP, entails the provision of ongoing support to teachers throughout their professional trajectories, facilitating the implementation of subject-specific teaching concepts. However, for such initiatives to be effective, it is imperative that the design of TPDPs incorporates key elements that authentically reflect the reality of teachers’ teaching practices. The subsequent section endeavors to furnish a synopsis of fundamental components that comprise effective TPDP.

### 4.1 Characteristics of effective TPDP

Lipowsky and Rzejak (2015, 2019) systematically reviewed the extant literature on TPDP, encompassing meta-analyses and reviews of pertinent studies. This comprehensive review yielded 10 characteristics that have been identified as essential to the efficacy of TPDPs. The characteristics enumerated below are thoroughly reflected upon and taken into account in the design of the MaTeGnu TPDP presented here.

1. Integration of the input, testing, feedback, and reflection phases
2. Relationship between the duration of TPDP and its effectiveness
3. Focus on subject-specific content and the subject-specific learning processes of students
4. Orientation toward findings from teaching research
5. Incorporation of scientific expertise
6. Opportunities to experience one’s own effectiveness
7. Think big, but start small
8. Provision of feedback and coaching for teachers
9. Promotion of cooperation among teachers
10. Situated learning through working with cases and examples from teaching practice.

### 4.2 Offer-and-use model for TPDP

The utilization of TPDPs to foster the advancement of professional competencies and the transformation of pedagogical practices constitutes a multifaceted endeavor. A multitude of conditions and factors exert a substantial influence on the transfer process and the efficacy of TPDP. In consideration of the aforementioned 10 characteristics of effective TPDP and drawing upon extant literature on TPDP as well as personal experience with TPDPs, Lipowsky developed the offer-and-use model for TPDP. The model delineates five categories of influencing factors that affect the transfer process (Lipowsky and Rzejak, 2015, 2019). It is noteworthy that the transfer process is only alluded to by Lipowsky and Rzejak (2015, 2019) and is not thoroughly elaborated upon. In the following list, the five categories of influencing factors mentioned by Lipowsky and Rzejak (2015, 2019) are enumerated and briefly explained.

**Offer: quality and quantity of learning opportunities during TPDP:** The offer-and-use model prioritizes the quantity and quality of learning opportunities within a TPDP course. These opportunities are determined by various factors, including the structural, didactic, and technical components of the course.

**Characteristics of the teacher educators:** The quality of the offerings is largely determined by the teacher educators’ knowledge, convictions, ability to motivate, ability to convincingly convey the relevance of the content to the participating teachers, motivational skills and other aspects of the teacher educator’s personality.

**Use: perception and utilization of learning opportunities by participating teachers:** However, the efficacy of a TPDP is contingent, to a considerable extent, on the manner in which the program’s offerings are utilized and processed by the participating teachers.

**Characteristics of participating teachers:** This process is influenced by a combination of motivational, social, personality-related, and cognitive prerequisites inherent to the participating teachers (Kennedy, 2016; Lipowsky, 2009; Santagata et al., 2010).

**School context:** Research has demonstrated that the professionalization process of teachers and, consequently, student learning are influenced by factors related to the school context (see Robinson and Timperley, 2007; van Veen et al., 2012), including direct and indirect support of teachers by school management and colleagues, cooperation structures, and the coherence of TPDP content with the current needs of school teaching reality.

### 4.3 TPDP transfer process model

**Figure 3:**
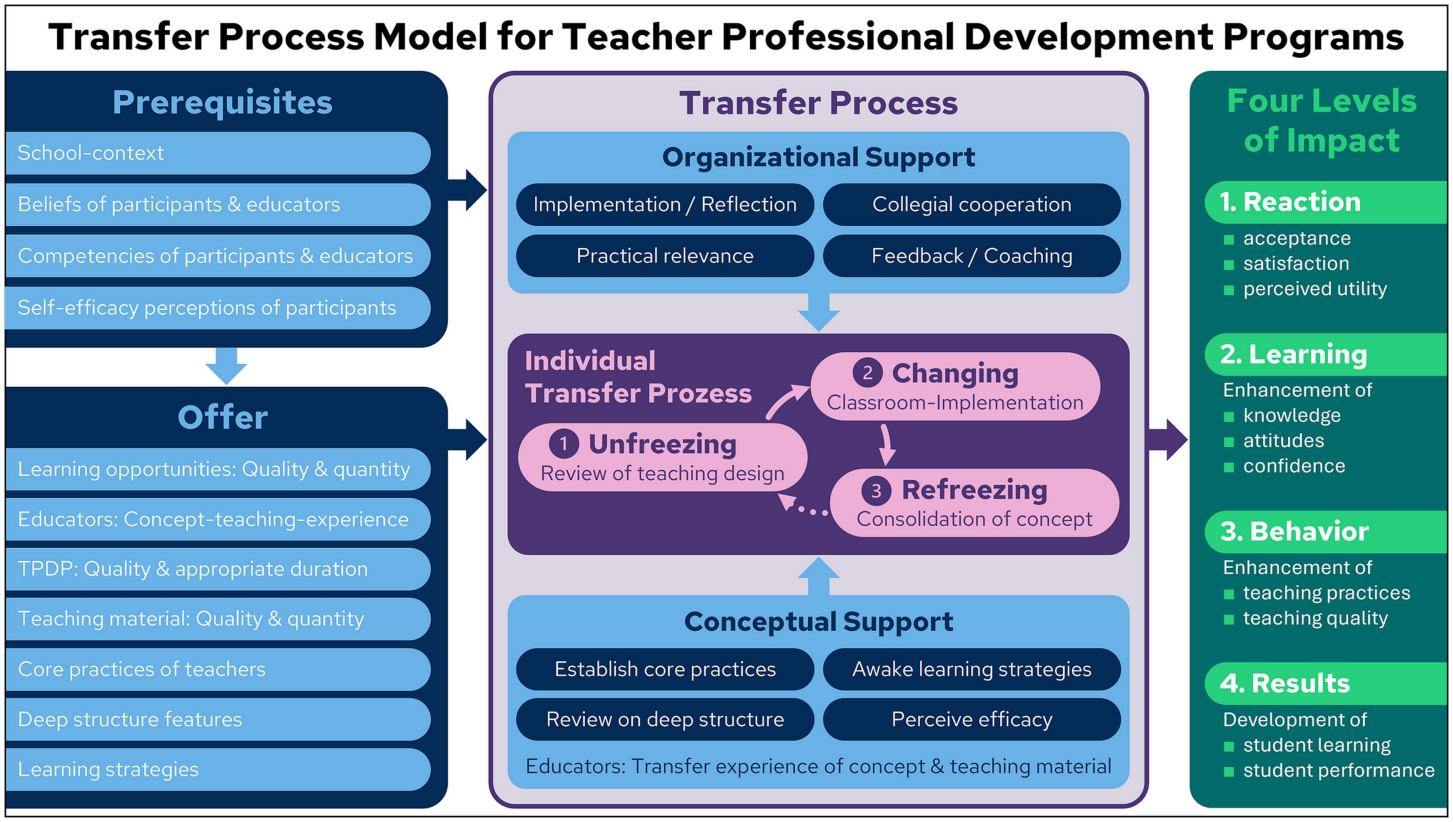
Transfer process model for teacher professional development programs (TPDP transfer process model): Inspired by concepts from Lipowsky and Rzejak (2015, 2019), Fütterer et al. (2024), and Kirkpatrick and Kirkpatrick (2016).

The key factors contributing to the success of TPDPs are described in the work of Lipowsky and Rzejak (2015, 2019), who provided their offer-and-use model as a foundational framework for understanding the influencing factors and success metrics in this domain. However, the pivotal transfer process itself is only referenced in Lipowsky and Rzejak’s (2015, 2019) work, and not thoroughly discussed. In the ensuing discourse, we propose a *transfer process model for teacher professional development programs*, which we refer to as the *TPDP transfer process model* (see Figure 3). It aims to answer the important question of how this process should be designed to implement the most effective TPDP. The TPDP transfer process model consists of four interrelated content clusters, namely the *prerequisites*, the *offer*, the *transfer process*, and the *four levels of impact*. These four clusters and their chain of effects are described in detail below.

#### 4.3.1 Prerequisites for TPDP

The TPDP transfer process model illustrates the prerequisites that influence transfer process. With regard to the *school context*, the presence or absence of direct and indirect support from school management and colleagues, as well as the existence of cooperation structures, are crucial for the transfer process of TPDP. Additionally, aligning the content of TPDP with ongoing or planned school and teaching development projects is essential. The *beliefs* of the participating teachers and teacher educators regarding the core concepts covered in the program significantly influence the transfer process because they either promote or hinder constructive cooperation. The potential development processes are contingent, to a considerable extent, on the *initial level of competencies* exhibited by the participating teachers and teacher educators as well as the *participants self-efficacy perception*. However, as outlined briefly above, the school context exerts a direct influence on the transfer process and an indirect influence via the offers developed for the TPDP. This is due to the fact that the anticipated or already known aspects of the school context will be taken into account when developing the program.

#### 4.3.2 The offer within TPDP

The initiation of a productive transfer process necessitates the formulation of a bespoke TPDP as an *offer*. This encompasses *learning opportunities* that must be meticulously designed in terms of *quality and quantity*. To that end, the organization requires *teacher educators* who have already gained their own *teaching-experience with the training concept*. This approach is instrumental in ensuring the efficacy and *quality* of TPDPs, facilitating the identification of an optimal *program duration* that aligns with the program content and the intended processes. In order to design a successful transfer process, the program must also include *teaching materials* that are usable by the participating teachers, are of high *quality*, and are of an appropriate *quantity*. An insufficient amount of material may result in an inadequate grasp of the concepts, while an excessive amount could overwhelm the teachers, potentially hindering their ability to thoroughly work through the materials. The TPDP concept must encompass emphasis on *core practices of teachers*, a focus on the *deep structure* of the intended teaching content, and the planned consideration of *learning strategies*. It is evident that these aspects constitute the *offer* incorporated into the transfer process that the participating teachers must undergo in order to achieve levels of impact for themselves and their students.

#### 4.3.3 The transfer process of TPDP

The majority of the prerequisites for and aspects of offers to TPDP, as outlined in Chapters 4.4.1 and 4.4.2, have been previously mentioned in the offer-and-use model proposed by Lipowsky and Rzejak (2015, 2019). In their model, Lipowsky and Rzejak (2015, 2019) clearly emphasized that all of these factors influence the transfer process and that the effects of TPDP are predominantly moderated by the transfer process. Consequently, the absence of any substantive discussion on the transfer process and its design in this model is particularly noteworthy. Our objective is to address this evident gap in the offer-and-use-model and to propose a solution through the implementation of the *TPDP transfer process model*. As demonstrated in Figure 3, the core of this model is constituted by a detailed sub-model of the transfer process.

The focal point of this sub-model is the *individual transfer process* of each participant in a TPDP. Teachers encounter novel knowledge and concepts within the framework of TPDP. As Fütterer et al. (2024) note, drawing on Lewin’s (1947) work, incorporating these elements into one’s own teaching practice necessitates an *individual transfer process* that can be conceptualized in three phases: (1) *unfreezing*, (2) *changing*, and (3) *refreezing*. In the initial phase, teachers meticulously *review* their *lesson design*, with a focus on targeted learning objectives (*unfreezing*). In the subsequent phase, teachers endeavor to reconceptualize their instructional design, revising its practical *implementation within the classroom* setting. If deemed beneficial, these modifications are implemented, albeit in a limited capacity, within the classroom environment (*changing*). This phase is frequently reiterated and characterized by a process of trial and error. Once the practical results are perceived as satisfactory, the *concept is consolidated* (*refreezing*).

The transfer process, initiated during the TPDP, contains two mechanisms of support for the individual transfer processes of the participating teachers. The first is *organizational support*, and the second is *conceptual support*.

*Organizational support* is characterized by a well-designed framework that enables the *implementation* of TPDP concepts in participants’ classes and facilitates systematic reflection on experiences gained throughout the process. Consistent *collegial cooperation* must be demanded and structurally supported. This builds and consolidates small teams of participants who experience the *practical relevance* of TPDP content together and encourage each other. In these professional learning communities, participants provide each other with *feedback* and avail themselves of *coaching* from accompanying teacher educators when necessary.

The provision of *conceptual support* is primarily undertaken by the teacher educators, who are required to possess the necessary qualifications for this role, typically attained through completion of an appropriate TPDP process. On the one hand, the teacher educators acquire experience with the teaching materials provided in the TPDP course by utilizing them in their own lessons and developing them further in a team setting so that they are suitable for teaching. Conversely, they acquire experience in imparting the fundamental principles of the TPDP by first acquiring these principles from experts and subsequently disseminating them in a more limited setting. In order to provide conceptual support, it is necessary to *establish core practices*, *awaken learning strategies*, *review on deep structure*, and *perceive efficacy*. The concept of core practices is elucidated by Shure et al. (2025), who define core practices for teaching as the recurring actions and decisions through which teachers address common challenges in instruction. Within the TPDP transfer process model (see Figure 3), two core practices of teaching are of particular importance: (1) the selection and adaptation of tasks, media, and representations, and (2) the observation and evaluation of student thinking.

In summary, the transfer process for TPDPs, as outlined in this chapter, encompasses two aspects: first, individual transfer processes among all participants, and second, a dual support system comprising organizational and conceptual support.

#### 4.3.4 The four levels of impact generated by TPDP

The transfer process, which is organized within the context of TPDP, can result in impact at four distinct levels. These levels of impact are consistent with Kirkpatrick’s four levels of training evaluation (Kirkpatrick and Kirkpatrick, 2016) which can be interpreted as attainable outcomes. In the following segments, we delineate and substantiate them for the TPDP transfer process model (see Figure 3) in accordance with the perception of these levels by Lipowsky and Rzejak (2015, 2019).

**Level 1 – Reaction:** The initial level, designated “Reaction,” focuses on the extent to which the participating teachers regard the TPDP as beneficial (*perceived utility*), appealing (*satisfaction*), and relevant to their daily teaching practices (*acceptance*).

**Level 2 – Learning:** The second level, entitled “Learning,” describes the degree to which participants acquire or enhance the intended *knowledge*, skills, *attitude*, *confidence*, and commitment based on their participation in the TPDP.

**Level 3 – Behavior:** The third level, designated “Behavior,” delineates the extent to which participants implement the insights acquired during the TPDP in their own instructional practice in terms of *enhancement of teaching practices and teaching quality*.

**Level 4 – Results:** The fourth level, entitled “Results,” characterizes the degree to which a targeted *development* of students’ comprehension in terms of *student learning and student performance* occurs as a result of the TPDP, the implemented support, and the provided teaching materials.

These levels can be used to describe the possible effects as well as serve as a theoretical basis for evaluating TPDP.

The TPDP transfer process model (see Figure 3) is comprised of four distinct content clusters that are interconnected: *prerequisites*, *offer*, *transfer process*, and *four levels of impact*, as well as their chain of effects. The theoretical underpinnings of the MaTeGnu TPDP, the subject of the ensuing chapter, are thereby delineated.

## 5 The MaTeGnu implementation of the TPDP transfer process model

The project MaTeGnu is a comprehensive TPDP for all schools with upper secondary levels in the German state of Rhineland-Palatinate. This initiative is a collaborative effort between the Ministry of Education of Rhineland-Palatinate and the Chair of Mathematics Education for Secondary Schools at the RPTU University of Kaiserslautern-Landau. The latter is responsible for (1) designing the program and its organizational structure, (2) developing concepts and content based on current research findings in mathematics education, and (3) providing substantive input for TPDPs. This ensures that MaTeGnu is based on research on teaching and learning mathematics (Loucks-Horsley et al., 2010; Taylor et al., 2017; van Veen et al., 2012) and, on the other hand, that scientific experts are leading the development and design of the program (Timperley et al., 2007), two proven essential criteria for successful TPDP. The program endeavors to remediate the identified issues in upper secondary mathematics education by prioritizing the cultivation of conceptual understanding with basic mental models and leveraging technology-enhanced teaching. MaTeGnu is meticulously developed in terms of content and structure to address the prevalent challenges associated with TPDP, implementing the proposed TPDP transfer process model.

### 5.1 Organizational support in MaTeGnu

In order to foster teaching development through TPDP in a systematic manner, it is essential to engage all relevant institutions. For MaTeGnu, this primarily signifies the Ministry of Education of Rhineland-Palatinate, which bears the primary responsibility for the province’s school education and exercises political control. Additionally, the State Institute of Education (in German: Pädagogisches Landesinstitut) must assume a central role, as it is responsible for TPDP in the state and maintains a group of especially trained teacher educators, designated as ATDs (advisors for mathematics teaching development). And finally, the supervision of mathematics instruction, the preparation and monitoring of upper secondary school graduation exams, and the respective TPDPs are the responsibility of the state’s six MCs (mathematics consultants). To employ and reinforce this TPDP network, the group of MaTeGnu teacher educators is composed of ATDs and MCs (see Chapter 5.1.2).

To ensure the coordinated development of mathematics teaching as a whole it is also imperative to incorporate the two other phases of teacher education beside the third phase (TPDP). MaTeGnu integrates the initial phase (pre-service university teacher education) through the RPTU project lead and the second phase, through the participation of three SLTs (subject leaders in mathematics at teacher training seminars) in the MaTeGnu expert team responsible for the iterative implementation and maturing of the MaTeGnu teaching material (see Chapter 5.1.2).

#### 5.1.1 MaTeGnu organizational prerequisite: school context

A central prerequisite for TPDP, as delineated in the TPDP transfer process model, is the school context (see Chapter 4.4.1). While it may appear to be of minor consequence, it in fact encompasses several elements that have the potential to impede the efficacy of teacher training programs. For example, the absence of a suitable mathematics class will prevent the immediate transfer process, the paucity of support of colleagues and administration will complicate the participation in the TPDP events. A prerequisite for every school to participate in MaTeGnu is the endorsement of the school administration and the mathematics department for the project and the teacher tandem, supporting the *collegial cooperation* on school level. The endorsement includes the continuous allocation of mathematics classes to these teachers throughout the 3 years of TPDP to ensure *practical relevance* for the participants and to enable immediate *implementation and reflection* of the TPDP content.

#### 5.1.2 MaTeGnu organizational prerequisite and offer: the teacher educators

The teacher educators engaged in the MaTeGnu program are already qualified teacher educators (MCs and ATDs) with considerable experience in TPDP. Thus, the MaTeGnu teacher educator qualification prioritizes the concept and content of the MaTeGnu project. To gain intensive experience with the MaTeGnu concept and teaching material, the central part of the teacher educator qualification is to participate in a complete three-year cycle of MaTeGnu TPDP (see Chapter 5.1.3) themselves. As participants they undergo the TPDP transfer process and assess the practicality of the MaTeGnu teaching material in a range of conditions through teaching according to MaTeGnu in their own upper secondary mathematics class (*Educators: concept-teaching-experience*).

Concurrently, the MaTeGnu expert team has been constituted responsible for the maturing of the teaching material. It comprises the MaTeGnu teacher educators, research-based university mathematics pre-service teacher educators from the MaTeGnu management team and the SLTs of the second phase of teacher education. The conceptual framework and teaching materials are refined in the group and elaborated based on the experiences in their individual transfer processes of the MaTeGnu TPDP cycle.

#### 5.1.3 MaTeGnu organizational offer and support: the MaTeGnu TPDP cycle

**Figure 4:**
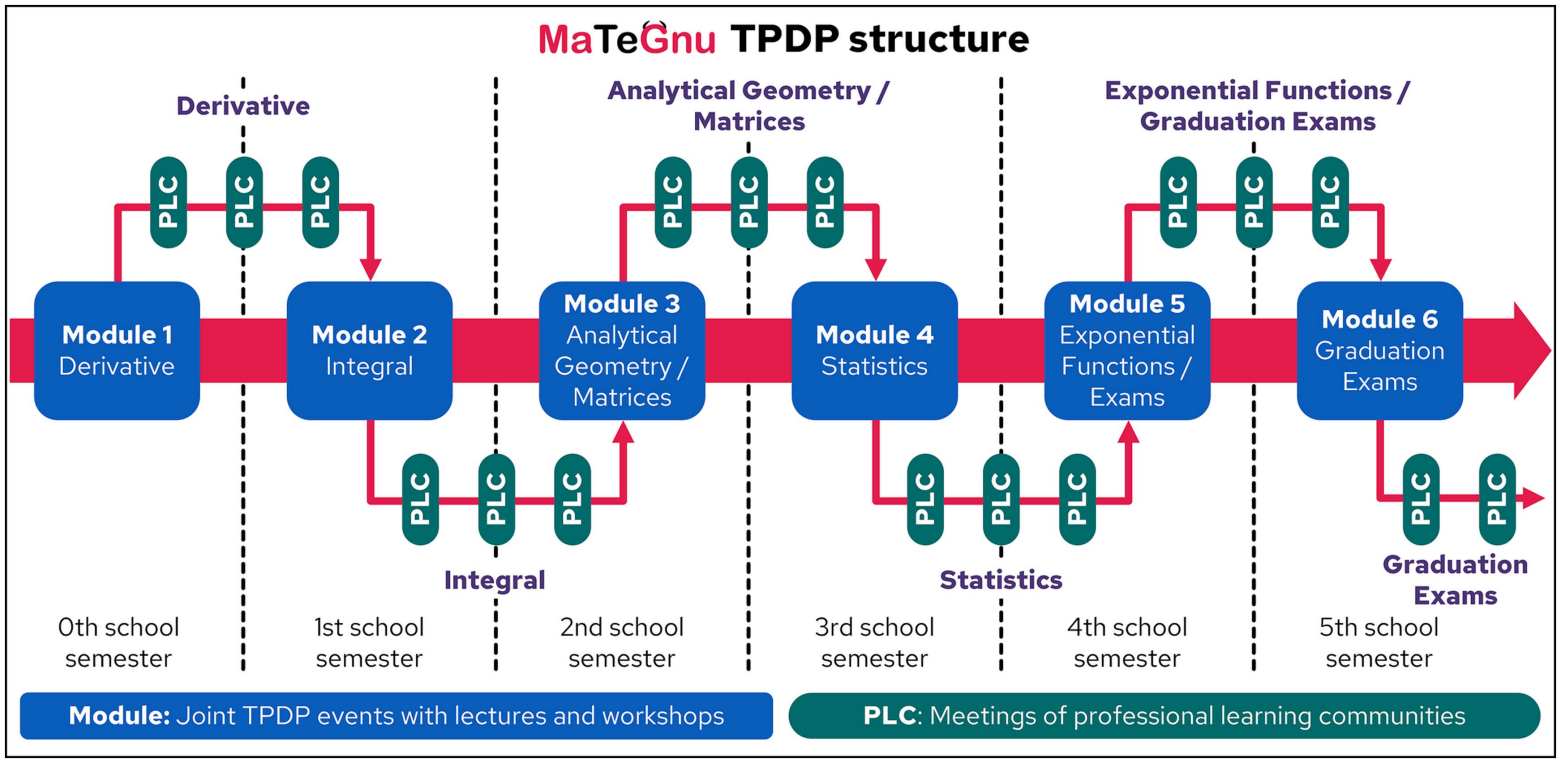
MaTeGnu TPDP structure.

The objective of MaTeGnu is to provide systematic support for teachers in upper secondary mathematics teaching. This encompasses a wide range of topics (derivatives, integrals, analytic geometry, matrix calculus, statistics, and exponential functions). For the necessary *focus on* subject-specific content and *student learning processes* in TPDP (Darling-Hammond et al., 2017; Timperley et al., 2007; van Veen et al., 2012) and for an *appropriate* relationship between the *duration* of the program and the desired goals (Timperley et al., 2007; Lipowsky and Rzejak, 2015, 2019), it is imperative that MaTeGnu TPDP encompass the entirety of the upper secondary school cycle, which entails a duration of 3 years. Hence, participating teachers are literally accompanied with their upper secondary school mathematics class through the complete cycle over 3 years (*implementation*; *practical relevance*; *appropriate duration*). Figure 4 illustrates the MaTeGnu program cycle’s structure, meticulously aligned to the mathematics curriculum (*practical relevance*). Over the course of these 3 years, the participants are engaged in biannual full-day TPDP events focused on the MaTeGnu concept and teaching materials (*quality and quantity of learning opportunities*). They are administered by research-based university mathematics educators and establish the foundation for the subsequent implementation of MaTeGnu concept in the participants’ teaching during the ensuing school semester (*core practices of teachers*; *deep structure features*).

#### 5.1.4 MaTeGnu organizational support: professional learning communities

The heart of the MaTeGnu transfer process support is each regional professional learning community (PLC), organized and led by a MaTeGnu teacher educator. The PLCs address the subsequent pivotal factors conducive to efficacious TPDP (see Chapter 4.1):

- Integration of the input, testing, feedback, and reflection phases (Darling-Hammond et al., 2017)
- Opportunities to experience one’s own effectiveness (Guskey, 2002; Timperley et al., 2007)
- Provision of feedback and coaching for teachers (Kalinowski et al. (2019); Darling-Hammond et al., 2017)
- Promotion of cooperation among teachers (DuFour, 2004; Lomos et al., 2011; Darling-Hammond et al., 2017; Kennedy, 2016)
- Situated learning through working with cases and examples from teaching practice (Richter et al., 2013; van Veen et al., 2012)

Within these PLCs, MaTeGnu teachers (1) seek support for and exchange ideas about the implementation of the concept and teaching material (*coaching; collegial cooperation*), (2) discuss (*feedback*) and reflect collectively on their teaching experiences and their transfer process (*implementation/reflection*), (3) identify best practice and analyze student interaction (*perceive efficacy*) and (4) receive specific support from MaTeGnu teacher educators (*feedback*/*coaching*). The deployment of MaTeGnu teacher educators as leaders of the PLCs is contingent upon the attainment of intensive teaching experience with the concept and materials in their own TPDP cycle (*Educators: concept-teaching-experience*).

### 5.2 Conceptual support in MaTeGnu TPDP

In addition to the previously delineated organizational support, the MaTeGnu TPDP concept encompasses a comprehensive array of measures designed to furnish conceptual support for the individual transfer process of the participating teachers.

#### 5.2.1 MaTeGnu conceptual offer and support: TET core practices of teachers

Participation in a TPDP focused on TET for the development of fundamental mathematical understanding has been shown to significantly enhance instructional practice, facilitated by collaboration (Konstantinidou and Scherer, 2022). The MaTeGnu concept is predicated on the integration of a focus on conceptual understanding with the development of TET skills regarding the universal digital mathematics tool GeoGebra through a combination of TPDP events and collaboration in PLCs. Specifically, the MaTeGnu events and PLCs aim to develop and consolidate *TET core practices* in the use of digital learning environments and GeoGebra with the following means:

1. Field-tested teaching material of digital learning environments (namely GeoGebra) with didactical TET implementation notes (see Chapter 5.2.2).
2. Accumulating workshops on *TET core practices* for the specific teaching material at the full-day events.
3. Accompanying monthly digital how-to and best-practice workshops [TET classroom practice (Simsek and Clark-Wilson, 2025) and noticing (McCulloch et al., 2023), technology-related *learning strategies of students* and technology-enhanced assessment], to provide customized support, catering to individual needs and objectives.

Since the integration of technology-related prior knowledge and beliefs amplify the efficacy of these programs (Bowman et al., 2022), MaTeGnu offers a one-year basic qualification already including the PLCs prior to MaTeGnu TPDP to integrate and balance TET knowledge, address TET-related beliefs and establish the PLCs (*prerequisites/offer: beliefs/competencies of participants*).

#### 5.2.2 MaTeGnu conceptual offer and support: teaching material

According to Dröse et al. (2025), combining material transfer strategies with TPDPs is the most promising approach when implementing instructional innovations (*implementation*). MaTeGnu TPDP provides teaching materials online as material packages, grouped according to the teaching modules (Module 1 derivatives: https://mategnu.de/m/l1e).

These material packages invariably contain the following (*teaching material: quality and quantity*):

1. Instructional grid with phases of teaching sequence, TET and BMM notes and clear reference to the mathematics curriculum. This facilitates rapid access to ideas and concepts, enabling teacher educators in the PLCs to underscore the significance of critical phases for conceptual understanding.
2. Didactical TET and BMM implementation notes for each instructional phase, providing a concise overview of fundamental structural elements and guidance on methodological design (incl. *awaking learning strategies for students* working with the digital learning material).
3. Digital learning material: worksheets, GeoGebra applets, and digital learning environments (adaption to own teaching is established as *TET core practice*).
4. References to compatible supplementary material in mathematics textbooks and digital textbooks.^1^^,^^2^

The packages have been designed and developed by the mathematics education research group at Chair of Mathematics Education for Secondary Schools at the RPTU University of Kaiserslautern-Landau and have matured in the expert team based on the diverse implementation processes of the MaTeGnu teacher educators. Through this collaborative adaptation and revision process, MaTeGnu teacher educators, are uniquely positioned to provide conceptual support on the materials and their diverse implementation in the classroom (*educator: transfer of concept and teaching with material*).

**Figure 5:**
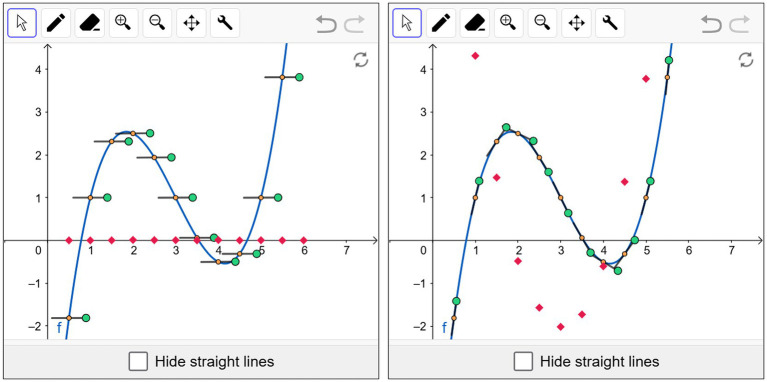
GeoGebra applet that displays tangential segments on the graph of the function f: left initial state, right target state (https://mategnu.de/m/l1e).

The following section exemplifies the learning material with a dynamic GeoGebra applet from a worksheet for graphing the derivative function, utilizing the basic mental model of derivative as tangent slope (see Chapter 2.4). As illustrated in Figure 5, it displays minute line segments alongside the function graph that can be tangentially aligned by dragging the green endpoints. The movement of the line segments is accompanied by the movement of the small red squares representing the slope of the tangential segment at the orange point. The configuration of the red squares provides an effective delineation of the graph of the derivative to f. The students can subsequently draw an approximation of the graph of the derivative function with the pencil tool.

#### 5.2.3 MaTeGnu conceptual offer and support: BMM as deep structure features

An essential aspect of the conceptual support provided by MaTeGnu is to familiarize teachers with the essential basic mental models, as well as to provide them with a meaningful sequence and weighting for addressing the relevant basic mental models. Regarding the basic mental models of derivatives, this signifies the establishment of conceptual understanding based on the local rate of change (RC) (see Chapter 2.4) and subsequently the graphical interpretation with the tangent slope (TS). There are several advantages to approaching the concept of derivation as a transition from the average rate of change to the local rate of change. Firstly, the kinematic context is part of young people’s everyday experiences (e.g., road traffic, computer games and sports). Furthermore, changes in speed over time are conceptually transparent to students and facilitate the understanding of the concept of the second derivative (acceleration). Therefore, the fundamental mental model of the derivative as the local rate of change (RC) serves as an effective starting point for the conceptual understanding of derivatives and facilitates their application to reality.

## 6 Evaluation of MaTeGnu

As delineated in Chapter 4.3, the TPDP transfer process model is to be employed in the evaluation of the MaTeGnu project, with the objective of encompassing all four Kirkpatrick levels. The present study is situated on the fourth level of training impact, which is the furthest remote from the training itself and seldom evaluated in studies concerning the effectiveness of training (Arthur et al., 2003; Stahnke et al., 2025). The objective of this study is to develop conceptual understanding among upper secondary school students of MaTeGnu trained teachers as a result of and concerning the major aim of MaTeGnu TPDP, which is to foster understanding with a focus on basic mental models.

The present study investigates the conceptual understanding of derivatives in relation to the utilization of the basic mental models of derivatives (see Chapter 2.4) and formal expression of derivatives. Initially, we seek empirical evidence for the supportive role of basic mental models in conceptual understanding:

*RQ1*: Do preferences for the utilization of basic mental models or formal expressions of derivatives relate to the understanding of the concept of derivatives?

To evaluate the actual effects of the TPDP on the students’ learning, a comparison was made between students in mathematics classes participating in MaTeGnu and those in regular mathematics classes regarding their preferences for the utilization of basic mental models or formal expressions and their conceptual understanding in the topic of derivatives:

*RQ2a*: Do students in MaTeGnu mathematics classes show different preferences to use explanations of derivative with basic mental models or formal expressions than students in regular mathematics courses?

*RQ2b*: Does the understanding of the concept of derivatives of students in MaTeGnu mathematics classes differ from that of students in regular mathematics classes?

Level four is the most remote in the effect chain of the TPDP. Considering the complex transfer process and the numerous factors influencing it, as delineated in the TPDP transfer process model (Figure 3), rather low effects are to be assumed for RQ2a and b. Nonetheless, it is a central objective of MaTeGnu with the three-year perspective as well as intensive peer interaction and coaching network to initiate, accompany and foster the transfer process.

## 7 Methodology

To address the research questions, a test on conceptual understanding of derivatives (CUD) is administered in conjunction with a test on basic mental models of derivatives (BMMD). These instruments are utilized to evaluate the learning outcomes of students following a series of lessons on derivatives. The combined test (CUD, BMMD) is delineated in a multi-matrix design, comprising a total of 58 items, which are distributed across six distinct booklets with 14 anchor items. Each booklet comprises 11 tasks, of which four are designated as anchor tasks. The test is scheduled to last for a duration of 45 min.

In order to address RQ1 we conducted correlation analyses of both test results using item response theory (IRT) with multi-dimensional Rasch models (see Chapter 7.3 for details). For RQ2a and b, an experimental-vs.-control-group design is employed, wherein the experimental group consists of students, who were instructed by MaTeGnu teachers according to the MaTeGnu concept. The control group consists of students from other mathematics classes at the same schools.

### 7.1 Test on conceptual understanding (CUD)

The FALKE2 test (accessible at Klinger, n.d.) serves as the foundation of our assessment of conceptual understanding of derivatives (CUD). FALKE2 is part of an evaluation framework for conceptual understanding in the domain of functions and early calculus with a broad theoretical and empirical validation base (Klinger, 2018, 2021). It is constructed as a post-test based on an overarching classification model consisting of three dimensions of conceptual understanding of functions:

1. LAYOUT, as the ability to use different representations (graph, situation, symbol) and to translate between them in a flexible way,
2. LAYER, as the mental images of functions, which are the basic mental models of functions (correspondence, covariation and object) as well as the action, process and object conceptions of a function (for details see Digel and Roth, 2022), and
3. LEVEL, as the handling of a stand-alone function or a function in conjunction with a transformation of the function or a function in conjunction with its derivative.

FALKE2 is validated as one dimensional construct and shows good reliability measures in the validation study (*α_C_* = 0.79 and *rel_EAP/PV_* = 0.79; Klinger, 2018, 2021). For the CUD, only those of the 26 items in FALKE2, that address the LEVEL derivative and at least one of the LAYERS are selected. Items that exclusively target procedural knowledge are excluded (e.g., state the equation of the derivative for a given functional equation). Table 1 lists all selected items with their content, item type, aspects of the 3L-Framework, and their solution rates in the validation study (Klinger, 2018, 2021).

**Table 1:** Selected items of FALKE2.

Item	Content	Type	Solution rate pilot	3L-framework Layout-Layer-Level	CUD booklet	CUD subscale
J9SE	FillVesselDraw	OA	48.6	SG-CV-F(D)	anchor	SIT
Y2VKa	Airplane	MCSS	69.4	SG-C-D	1; 4; 5	SIT
Y2VKb	Airplane	MCSS	67.6	SG-C-D	1; 4; 5	SIT
Y2VKc	Airplane	MCSS	35.5	SG-C-D	1; 4; 5	SIT
W7CK	ZoomIn	MCSS	74.7	G-O-FD	1; 4; 5	SIT
Z8PCa	SignDeriv	MCMS	18.4	GF-CVO-FD	1; 4; 5	GF
Z8PCb	SignDeriv	MCMS	25.8	GF-CVO-FD	1; 4; 5	GF
Z8PCc	SignDeriv	MCMS	37.4	GF-CVO-FD	1; 4; 5	GF
Z8PCd	SignDeriv	MCMS	15.7	GF-CVO-FD	1; 4; 5	GF
Z8PCe	SignDeriv	MCMS	20.8	GF-CVO-FD	1; 4; 5	GF
D6LGa	DerivParab	MCSS	86.9	GF-CV-FTD	2; 3; 6	GF
D6LGb	DerivParabReason	OA	37.0	GF-CV-FTD	2; 3; 6	GF
X4TP	GraphDerivSel	MCSS	35.7	SG-O-FTD	1; 4	GD
U3TP	GraphDeriv	OA	73.3	G-CV-FD	anchor	GD
V3RKa	GraphDerivMap	MCMS	56.5*	G-CV-FD	2; 3; 6	GD
V3RKb	GraphDerivMap	MCMS	56.5*	G-CV-FD	2; 3; 6	GD
V3RKc	GraphDerivMap	MCMS	56.5*	G-CV-FD	2; 3; 6	GD
S3AB	GraphDerivDraw	OA	43.6	G-CV-FD	2; 3; 6	GD

LAYOUT S, Situation; G, Graph; F, Formal-symbolic. LEVEL F, stand-alone Function; T, Function and Transformation; D, Function and Derivative; LAYER C, Correspondence; V, Covariation; O, Object. CUD, Test on conceptual understanding of derivatives (booklets 1–6); CUD Subscale SIT, situational context; GF, formal-symbolic with graph; GD, graphical derivation. *Rated in the validation study as one single item.

**Figure 6:**
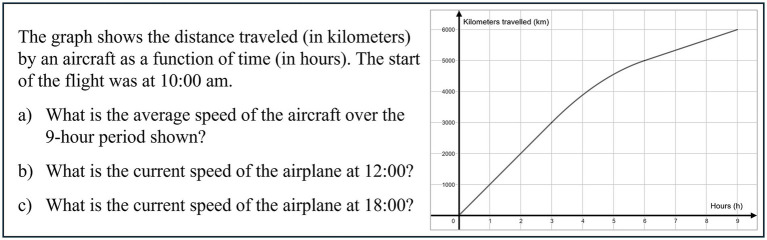
Task Y2VKa-c of FALKE2 test was translated from Klinger (n.d.), https://www.falke-test.de/, CC BY-NC-SA 4.0.

The prompt in task Y2VKa-c (see Figure 6) offers a graph representing the distance of a plane from its origin as a function of the duration of the flight and a description of the situation.

In item Y2VKa, students are tasked with estimating the mean velocity of the plane over the entire flight period displayed. They are also required to determine the instantaneous velocity at two distinct times in items Y2VKb and c. All three items aim at the LAYOUT situation and graph, the LEVEL function and derivative, and the LAYER covariation. The task can also be discussed from the perspective of basic mental models of derivatives, in particular the local rate of change. Determining the mean velocity (Y2VKa) demands an understanding of the mean rate of change. Consequently, students can apply the concept of rate of change as a relative size of two quantities, whereas for the instantaneous velocity (Y2VKb d c), as the local rate of change requires an understanding of rate of change as a limit of quotients (Byerley et al., 2012).

### 7.2 Test on basic mental models (BMMD)

Greefrath et al. (2021) have developed a test to assess students’ characteristics of basic mental models of derivatives (GV-A, accessible at https://hal.archives-ouvertes.fr/hal-03103685) with an empirically separate, reliable scale for each basic mental model (Cronbach’s alpha *α_C_* = 0.76 RC/0.79 TS/0.86 LL/0.90 AF; Greefrath et al., 2023). Each of the 13 tasks presents a mathematical situation (four of them set in a context) and offers four correct explanations, one corresponding to each basic mental model. The students are asked to rate the accordance of each explanation with their own thinking on a unipolar five-point Likert-Scale. To contrast the four understanding-based explanations we add an explanation with a formal-symbolic focus (FS) to each task. Due to limited test time, we selected eight tasks for our test on basic mental models of derivatives (BMMD). Following the idea of different LAYOUTS in FALKE2 we included all three tasks without graphs in the prompt and with respect to different LAYERS eliminated tasks with identical content focus (i.e., explanation of the situation at the inflection point of a graph) and finally balanced the number of inner-mathematical and contextualized items.

**Figure 7:**
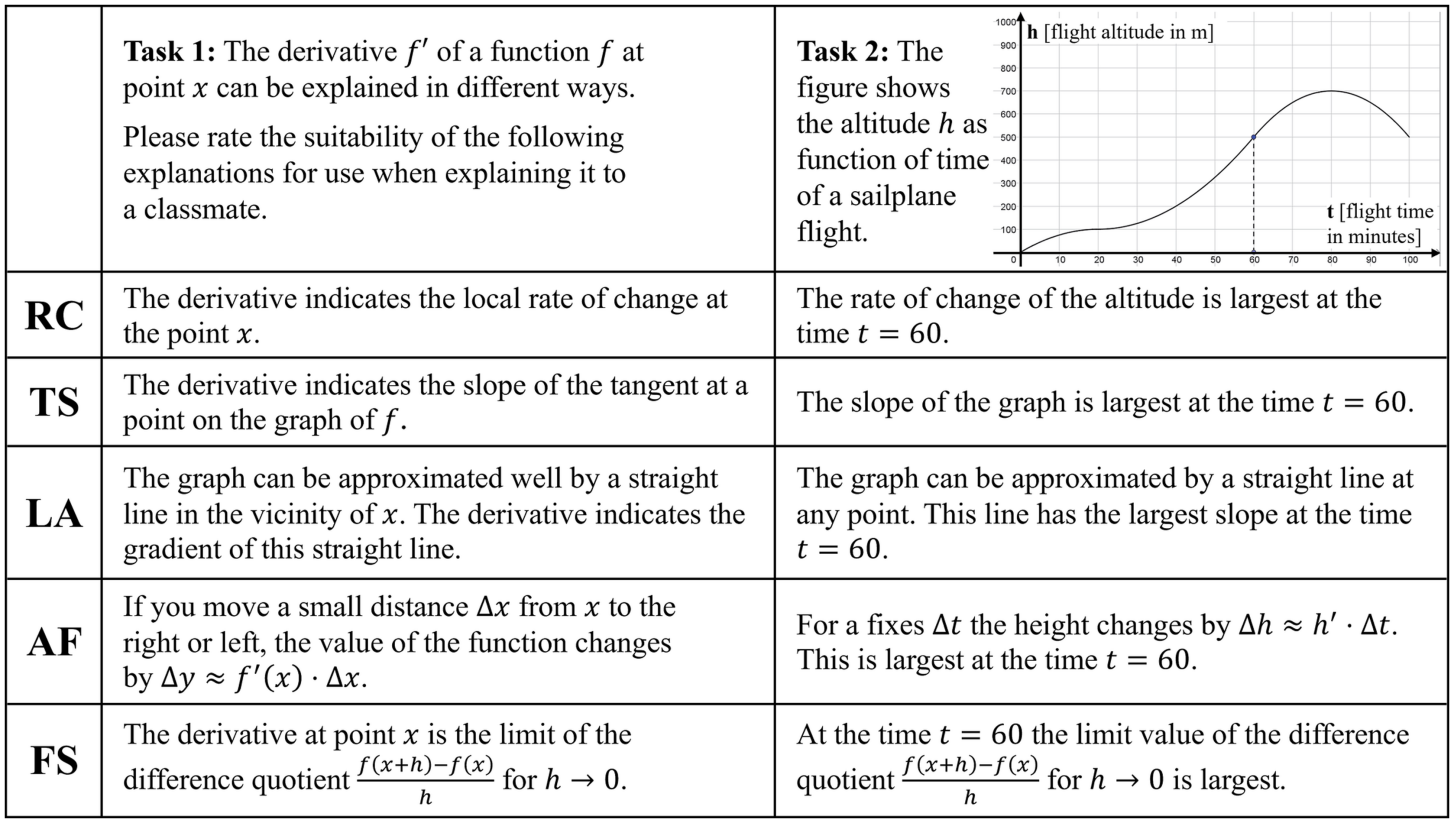
Sample tasks (inner-mathematical left, contextualized right) of BMMD. RC, local rate of change; TS, tangent slope; LL, local linearity; AF, amplification factor; FS, formal-symbolic.

In line with the understanding of basic mental models as mental representations that enable operative action on the level of thought and recognition of mathematical structures within contexts (Hefendehl-Hebeker et al., 2019), we modified the prompts in the GV-A towards a utilization perspective of basic mental models, asking the students to rate the suitability of the explanations (corresponding to the basic mental models RC, LL, TS, AF, and formal-symbolic FS) for their own explanation of the situation to a classmate. Finally, to force a decision we use a four-point Likert-Scale (see Figure 7 for an inner-mathematical and a contextualized sample task of BMMD).

### 7.3 Data analysis

The open answer items in the CUD test are coded with the solution criteria provided by the FALKE2 code manual and transferred to a dichotomous rating using predefined criteria thresholds (Klinger, 2018). The responses provided in BMMD (Likert-Scale) are then aggregated to form dichotomous coding (1 = suitable, 0 = not suitable). The dichotomous data from both tests is analyzed using IRT to answer RQ1. For RQ2a, the four-point Likert-Scale of the BMMD is assumed to be an interval scale (4 = very suitable to 1 = not suitable) in accordance with the validation study of the GV-A (Greefrath et al., 2023) to calculate the descriptive statistics for the five subscales (RC, TS, LL, AF, and FS) over all eight tasks. To determine differences between EG and CG we perform a Welch-Test on the individual mean values (as individual rating score instead of sum scores) of the distinct subscales of BMMD for RQ2a and the person ability scores in CUD from the Rasch model for RQ2b (Rasch et al., 2011). All analyses were conducted using R.^3^

### 7.4 Study sample

The sample of the evaluation study presented in this paper is drawn from the MaTeGnu teacher educator qualification (see Chapter 5.1.2). 14 ATDs and 4 MCs participated in the MaTeGnu TPDP with their mathematics classes (8 advanced/7 basic mathematics classes, 12 grammar/3 comprehensive schools). The students of these classes underwent a combined test (CUD, BMMD) after completing their MaTeGnu lesson series on derivatives and formed the experimental group (EG). This subsample consists of *N_EG_* = 151 students (76 female, 75 male, 0 diverse) with an average age of *M* = 17.0. Data was collected from June to December 2024.

Prior to the start of the MaTeGnu teacher educator qualification, 45 mathematics classes (10 comprehensive/35 grammar school classes, 23 advanced/22 basic mathematics classes) from the schools of the teacher educators participated in the combined test. This subsample forms the control group (CG) where no MaTeGnu TPDP treatment took place before and during teaching of derivatives. It consists of *N_CG_* = 571 students (276 female, 294 male, 1 diverse) with an average age of *M* = 17.1. Data was collected from June to November of 2023. The study was approved by the state school administration (ADD). All participants and, in the case of minors, their legal guardians, were informed of the study’s protocol and data confidentiality and gave their consent to participate.

## 8 Results

### 8.1 Correlation between understanding and basic mental models

The objective of this study was to investigate the relationship between the conceptual understanding of derivatives and the utilization of basic mental models. To this end, the dichotomous data from both tests (CUD, BMMD) from the entire data sample (experimental and control group together, *N* = 722) was analyzed using IRT. As one subscale of the BMMD is formed by formal-symbolic explanations and not based on basic mental models, the BMMD is split into two dimensions, resulting in a three-dimensional Rasch model with the concept test CUD on the first dimension, the subscales of all basic mental models (RC/LL/TS/AF) of the dichotomous BMMD on the second, and the formal-symbolic subscale (FS) on the third dimension.

The three-dimensional model shows good to acceptable reliability *rel_WLE-3_* = 0.80/0.74/0.70. There are moderate to weak correlations (Dancey and Reidy, 2020) between all three dimensions. The correlation *r* = 0.56 between conceptual understanding (CUD) and basic mental models (RC/TS/LL/AF) is significantly higher (Cohen et al., 2003) than the correlation *r* = 0.48 between formal-symbolic (FS) and understanding (CUD) (*z* = 2.471, *p* < 0.01) and the correlation *r* = 0.42 between formal-symbolic (FS) and basic mental models (RC/TS/LL/AF) (*z* = 4.426, *p* < 0.001).

For a closer look we separate the basic mental models of BMMD, with each model constituting a single dimension. The six-dimensional Rasch model (CUD, RC, LL, TS, AF, FS) demonstrates acceptable reliability (George and Mallery, 2019) for the whole data sample *rel_WLE-6_* = 0.79/0.69/0.72/0.70/0.68/0.70. Table 2 presents the latent correlations between the six dimensions of the Rasch model. The highest correlation was found between the dimensions tangent slope and local linearity (*r* = 0.72), followed by tangent slope and rate of change (*r* = 0.66), and the lowest between amplification factor and conceptual understanding (*r* = 0.32).

**Table 2:** Latent correlations of the six-dimensional RASCH-model of dichotomous CUD total scale BMMD (joint data of experimental and control group).

	CUD total scale	BMMD subscale RC	BMMD subscale TS	BMMD subscale LL	BMMD subscale AF	BMMD subscale FS
CUD	1	0.58	0.60	0.57	0.32	0.48
RC	–	1	0.66	0.56	0.38	0.36
TS	–	–	1	0.72	0.37	0.47
LL	–	–	–	1	0.40	0.45
AF	–	–	–	–	1	0.42

CUD, conceptual understanding of derivatives test; BMMD. basic mental models of derivatives test, with the scales; RC, local rate of change; TS, tangent slope; LL, local linearity; AF, amplification factor; FS, formal-symbolic.

While the three dimensions RC, TS and LL of basic mental models show significantly higher correlations (one-sided test; Cohen et al., 2003) to conceptual understanding (*r* = 0.58; *r* = 0.60; *r* = 0.57) than formal-symbolic (*r* = 0.48) to conceptual understanding (*z* = 2.995, *p* < 0.001; *z* = 3.949, *p* < 0.001; *z* = 2.862, *p* < 0.005), the correlation between the basic mental model dimension AF and conceptual understanding (*r* = 0.32) is significantly lower than between formal-symbolic and conceptual understanding (*z* = −4.489, *p* < 0.001). The basic mental model dimension amplification factor (AF) also exhibits significantly lower correlations to the other basic mental model dimensions (RC, TS, LL) than the correlations among these dimensions (e.g., LL ~ AF vs. LL ~ TS *z* = −10.345, *p* < 0.001; LL ~ AF vs. LL ~ RC *z* = −4.636, *p* < 0.001).

### 8.2 Basic mental models

The four-point Likert-Scale data of BMMD demonstrates good to questionable reliability (George and Mallery, 2019) across all subscales (RC, LL, TS, AF, FS) in both the control and the experimental group. Beside Cronbach’s alpha values for reliability Table 3 also displays the mean values and standard deviations for each subscale in the control and the experimental group and the results of the Welch-tests between both groups.

**Table 3:** Cronbach’s alpha reliability, mean value, standard deviation and effect size of the subscales of BMMD and iSubscale TS subscales in experimental (EG) and control group (CG).

	Subscale RC		Subscale TS		Subscale LL		Subscale AF		Subscale FS	
	CG	EG	CG	EG	CG	EG	CG	EG	CG	EG
α_C_	0.67	0.51	0.81	0.60	0.78	0.56	0.65	0.54	0.66	0.50
*M*	2.78	3.02	2.96	3.08	2.78	2.93	1.95	2.06	2.15	2.09
SD	0.92	0.85	0.97	0.89	0.88	0.85	0.89	0.84	1.04	0.94
Welch-Test	***t*(267) = 5.17 *p* < 0.001**		*t*(286) = 2.04 *p* = 0.021		***t*(295) = 2.81 *p* < 0.005**		*t*(570) = 2.23 *p* = 0.013		*t*(289) = −0.92 *p* = 0.179	
*d*	**0.474**		0.187		**0.258**		0.205		−0.084	

RC, local rate of change; TS, tangent slope; LL, local linearity; AF, amplification factor; FS, formal-symbolic. α_C_, Cronbach’s alpha reliability; *M*, mean value; SD, standard deviation; *d*, Cohen’s *d* effect size; significant effects are shown in bold.

Students in the MaTeGnu classes (experimental group) exhibit significantly higher utilization scores for the basic mental models (RC) rate of change (*t*(267) = 5.17, *p* < 0.001) and (LL) local linearity (*t*(295) = 2.81, *p* < 0.005) than students in the control group. The utilization scores of the basic mental models (TS) tangent slope (EG: *M* = 3.08, *SD* = 0.89; CG: *M* = 2.96, *SD* = 0.97) and (AF) amplification factor (EG: *M* = 2.06, *SD* = 0.84; CG: *M* = 1.95, *SD* = 0.89) are also higher in the experimental group, but not statistically significant. In contrast to the basic mental mo.els, the score of the formal-symbolic explanations (FS) in the experimental group is lower (EG: *M* = 2.09, *SD* = 0.94.CG: *M* = 2.15, *SD* = 1.04). Yet, the difference is not statistically significant (see Table 3 for further details).

A more thorough examination of the utilization scores across the different tasks reveals some distinctions. Table 4 presents the mean values of the subscales (RC, LL, TS, AF, FS) for the respective tasks of BMMD separately for both groups (CG, EG). There are higher utilization scores for the basic mental models in the contextualized tasks than in the inner-mathematical tasks.

**Table 4:** Mean value and standard deviation of the subscales of BMMD of aSubscale LL tasks in experimental (EG) and control group (CG).

		Subscale RC		Subscale TS		Subscale LL		Subscale AF		Subscale FS	
		*M*	SD	*M*	SD	*M*	SD	*M*	SD	*M*	SD
Task 2—GVAbWp9 Sailplane height inflection point	CG	3.09	0.91	3.27	0.91	3.07	0.93	2.04	0.87	2.03	0.97
	EG	3.34	0.67	3.28	0.82	3.18	0.88	1.91	0.78	2.03	0.80
Task 3—GVAbMax2 Cars in tunnel maximum	CG	2.48	0.91	2.99	1.05	2.86	0.94	2.03	0.86	1.99	1.27
	EG	2.51	0.80	3.06	0.97	2.92	1.00	2.18	0.87	2.01	0.97
Task 4—GVAbWp3 Bicycle path inflection point	CG	2.87	0.91	3.08	0.94	2.92	0.94	2.16	0.95	2.05	1.00
	EG	2.98	0.91	2.98	0.91	3.06	0.80	2.22	0.80	2.12	0.86
Task 7—GVAbfg12 number of bacteria	CG	2.82	0.86	3.28	0.84	3.09	0.86	2.22	0.96	1.95	0.92
	EG	3.25	0.80	3.36	0.76	3.17	0.89	2.34	0.91	1.81	0.84
Task 8—GVAbNd13 Explain with graphs not differentiable	CG	2.69	0.82	3.03	0.94	3.02	0.95	1.96	0.89	2.10	1.06
	EG	2.97	0.82	3.23	0.87	3.05	0.95	2.24	0.78	1.96	0.96
Task 1—GVAbStX1 Explain f’ (nG)	CG	2.83	0.92	3.09	0.95	2.58	0.98	1.67	0.79	2.22	1.08
	EG	3.15	0.88	3.14	0.83	2.67	0.87	1.74	0.80	2.23	1.02
Task 5—GVAbNd4 Explain not differentiable (nG)	CG	2.39	0.82	2.89	0.91	2.80	0.89	2.19	0.92	2.29	1.00
	EG	2.55	0.74	2.95	0.84	2.65	0.91	2.36	0.90	2.40	0.99
Task 6—GVAbDop10 Double f doubles f’ (nG)	CG	2.78	0.94	2.34	0.91	2.70	0.90	2.02	0.88	2.15	1.02
	EG	3.06	0.89	2.32	0.81	2.81	0.87	1.94	0.65	2.04	0.98

RC, local rate of change; TS, tangent slope; LL, local linearity; AF, amplification factor; FS, formal-symbolic. nG, no Graph in task presented; *M*, mean value; SD, standard deviation.

There are higher mean values for the basic mental models and at the same time lower for the formal-symbolic subscale in the contextualized tasks than in the inner-mathematical tasks, but the differences between control group and experimental group are not observably greater or smaller in contextualized tasks than in inner-mathematical tasks. In tasks than present graphs in the prompt the mean values for tangent slope and local linearity are higher.

**Figure 8:**
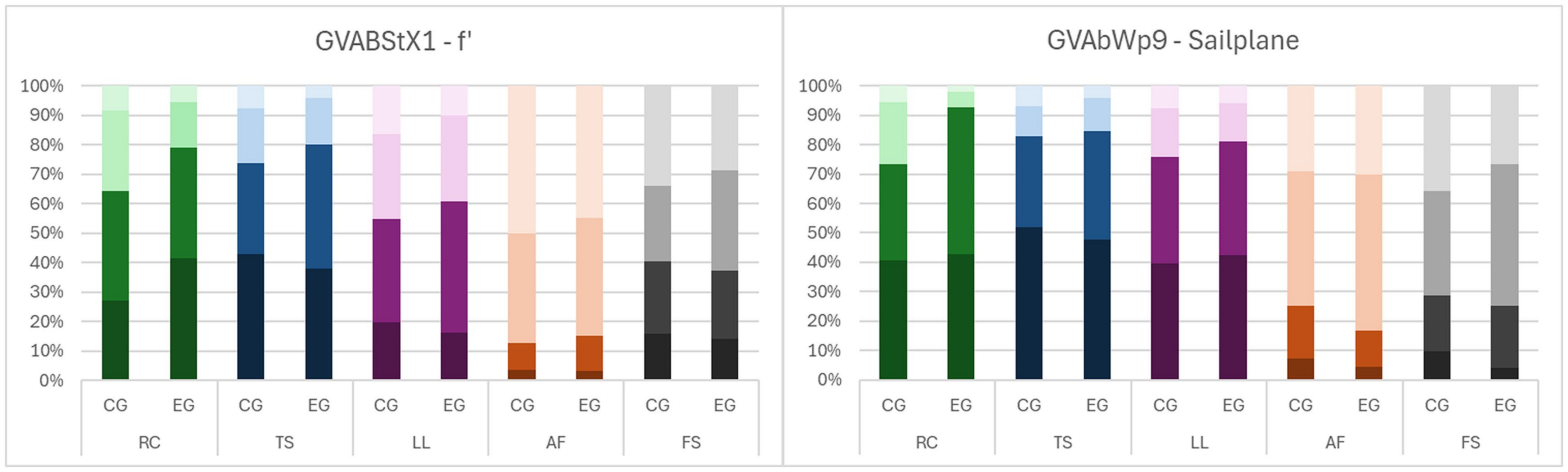
Rating distributions of two tasks (inner-mathematical left, contextualized right) of BMMD. RC, local rate of change; TS, tangent slope; LL, local linearity; AF, amplification factor; FS, formal-symbolic; EG, experimental group; CG, control group.

Figure 8 illustrates these patterns. It shows the distribution of ratings (as percentage) in all subscales (RC, LL, TS, AF, FS) for an inner-mathematical tasks (without a graph in the prompt) and a contextualized tasks (with a graph in the prompt). Darker colors represent higher ratings (very suitable, suitable, less suitable, not suitable) and the grey color is used to separate the formal-symbolic subscale (FS) from those of basic mental models.

**Figure 9:**
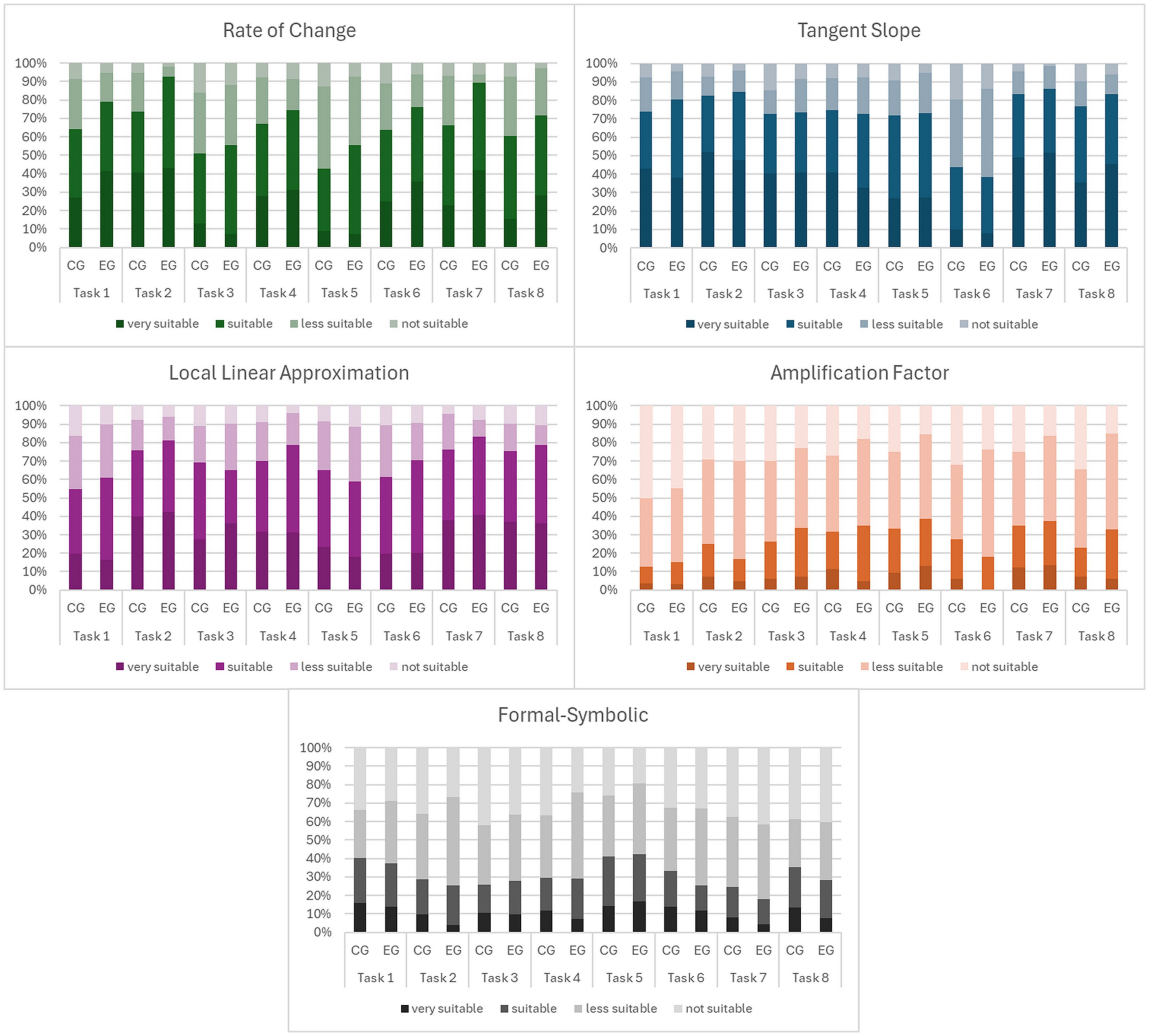
Likert-scale ratings of all tasks in control group (CG, left) and experimental group (EG, right) grouped by scales of BMMD.

There is a noticeable increase in positive ratings in the experimental group compared to the control group in both tasks for the three basic mental models local rate of change (RC), tangent slope (TS) and local linearity (LL) and a slight decrease for formal-symbolic (FS) explanations. The level of positive ratings in RC/TS/LL is higher and in FS lower in the contextualized task and in this task the differences between experimental and control group are greater. The level of positive rating for amplification factor is very low in both tasks and even drops in the experimental group in the contextualized task. Figure 9 gives an insight into all tasks of the BMMD. It displays all ratings grouped by the different scales.

The ratings of rate of change vary most, between tasks and between experimental group and control group. The ratings for the basic mental models and the formal-symbolic explanation vary over the different tasks, except for tangent slope, which has consistently high positive ratings in all tasks but task 6.

### 8.3 Conceptual understanding of derivatives

The dichotomous data of CUD shows acceptable to questionable reliability (George and Mallery, 2019) for the total scale and good to poor reliability for the subscales situational (SIT), formal (GF) and graphical derivation (GD) in the control and experimental group. Table 5 lists Cronbach’s alpha values for reliability, mean values and standard deviations for each subscale in the control and experimental group and the results of the Welch-tests between both groups.

**Table 5:** Cronbach’s alpha reliability, mean value, standard deviation and effect size of the CUD and its subscales in experimental (EG) and control group (CG).

	Total Scale		Subscale SIT		Subscale GF		Subscale GD	
	CG	EG	CG	EG	CG	EG	CG	EG
α_C_	0.57	0.60	0.70	0.80	0.46	0.55	0.65	0.50
*M*	5.03	6.06	1.54	2.27	1.66	2.07	1.84	1.69
SD	8.20	9.01	2.46	2.73	1.83	2.50	2.42	1.93
Welch-Test	***t*(225) = 3.78 *p* < 0.001**		***t*(225) = 4.92 *p* < 0.001**		***t*(210) = 2.93 *p* < 0.01**		*t*(257) = −1.08 *p* = 0.140	
*d*	**0.346**		**0.451**		**0.268**		−0.099	

CUD Subscale SIT, situational context; GF, formal-symbolic with graph; GD, graphical derivation. α_C_, Cronbach’s alpha reliability; *M*, mean value; SD, standard deviation; *d*, Cohen’s d effect size; significant effects are shown in bold.

Students in the MaTeGnu classes (EG) exhibit significantly higher conceptual understanding (*t*(225) = 3.78, *p* < 0.001, *d* = 0.346) than students in the control group (EG: *M* = 6.06, *SD* = 9.01; CG: *M* = 5.03, *SD* = 8.20) with a small to medium effect (Cohen, 1988). In the subscale (SIT), formed by the contextualized tasks of the CUD, the score of MaTeGnu students is significantly higher (EG: *M* = 2.27, *SD* = 2.73; CG: *M* = 1.54, *SD* = 2.46) with an almost medium effect (*t*(225) = 4.92, *p* < 0.001, *d* = 0.451). The difference between experimental and control group in the subscale with formal tasks, where a graph is presented (GF), is also significant (*t*(210) = 2.93, *p* < 0.01, *d* = 0.268) with a small effect size, but this result has to be interpreted with caution, since the reliability of the scale is not satisfactory (see Table 5). The scores in the subscale for graphical derivations is slightly lower in the experimental group (EG: *M* = 1.69, *SD* = 1.93; CG: *M* = 1.84, *SD* = 2.42), but the difference is not significant (*t*(257) = −1.08, *p* = 0.140) and the reliability of the subscale in the experimental group is not acceptable.

In comparison to the solution rates of the different tasks reported by Klinger (2018) in the validation study of the FALKE2-test, those in the control group and experimental group of our study are higher on average, but the pattern is similar (see Table 6). However, several items of task Z8PC and item D6LGb demonstrate considerably higher solution rates in our study than in the validation study.

**Table 6:** Solution rates of the CUD tasks in validation study (VAL), experimental group (EG) and control group (CG).

Item	Subscale	Solution rate VAL	Solution rate CG	Solution rate EG
J9SE	SIT	48.6	45.7%	53.1%
Y2VKa	SIT	69.4	71.4%	72.7%
Y2VKb	SIT	67.6	68.2%	75.5%
Y2VKc	SIT	35.5	34.4%	34.7%
W7CK	SIT	74.7	76.1%	84.8%
Z8PCa	GF	18.4	27.4%	40.4%
Z8PCb	GF	25.8	51.7%	47.5%
Z8PCc	GF	37.4	37.3%	61.6%
Z8PCd	GF	15.7	24.7%	34.3%
Z8PCe	GF	20.8	35.5%	49.5%
D6LGa	GF	86.9	83.8%	82.0%
D6LGb	GF	37.0	83.1%	78.4%
X4TP	GD	35.7	35.7%	44.3%
U3TP	GD	73.3	75.3%	70.7%
V3RKa	GD	56.5*	78.0%	82.4%
V3RKb	GD	56.5*	51.3%	47.1%
V3RKc	GD	56.5*	51.3%	47.1%
S3AB	GD	43.6	33.2%	40.0%

CUD Subscale SIT, situational context; GF, formal-symbolic with graph; GD, graphical derivation. *Rated in the validation study as one single item.

## 9 Discussion

The present study evaluates the effects of the TPDP MaTeGnu on student understanding of derivatives. As a first step we investigate the core assumption of the TPDP, to foster understanding with basic mental models (RQ1). Based on that we compare students whose teachers participate in MaTeGnu with their mathematics class and teach derivatives according to MaTeGnu (experimental group), to students from other mathematics classes (control group). We investigate their utilization of basic mental models and formal-symbolic explanations (RQ2a) as well as their conceptual understanding (RQ2b).

### 9.1 The relation of basic mental models and conceptual understanding

As delineated in Chapter 2.3, basic mental models provide content-related interpretations to abstract mathematical concepts, giving them a meaning. It seems more than feasible that meaningful acquisition of mathematical concepts should enhance understanding. But especially on high-school level the question remains whether students can make use of these meaningful concepts in mathematical tasks or if arguing on a formal-symbolic level is more profitable for solving the tasks.

The satisfactory reliability of the IRT modeling demonstrates the suitability of both test instruments for this study. The results of our correlation analyses of the Rasch-modelled dichotomous test-data (see Chapter 8.1) show a strong correlation between basic mental models and conceptual understanding (Cohen, 1988), also an indicator for the validity of both tests. The correlations between basic mental models and conceptual understanding are significantly higher than those of formal-symbolic explanations and conceptual understanding. Thus, students who prefer the use of basic mental models for explanations over formal-symbolic expressions show higher conceptual understanding of derivatives. Hence, we can assume that a focus on basic mental models when developing the mathematical concept of derivative fosters conceptual understanding.

But in our study not all basic mental models are strongly tied to conceptual understanding. The amplification factor (AF) shows low utilization scores in the BMMD and a weak correlation to understanding. AF is also only weakly related to the other basic mental models. This is in accordance with previous studies on the structure of students’ basic mental models (Greefrath et al., 2023), where explanations based on AF also showed the lowest agreement and AF was only loosely related to tangent slope (TS) and local linearity (LL), but opposed to our results AF showed a medium to strong correlation to rate of change (RC) in their study. The strong correlation between TS and LL is also a replication of the results in the validation study and can be explained by their broad common theoretical grounds as discussed by Tall (2012).

Regarding RQ1 we can conclude that preferences for the utilization of rate of change, tangent slope and local linearity are strongly related to the understanding of the concept of derivatives, whereas preferences for the utilization of amplification factor (AF) or formal-symbolic explanations (FS) only play a subordinate role for conceptual understanding.

The results provide empirical support for the concept of MaTeGnu, especially its focus on the deep structure of teaching (TPDP transfer process model, see Figure 3) in two ways. First, they underpin the central aim of MaTeGnu to foster conceptual understanding through the development of basic mental models. Second, the subordinate role of amplification factor for conceptual understanding and the high correlation between local linearity and tangent slope sustain the MaTeGnu focus on rate of change and tangent slope for the concept of derivatives (see Chapter 5.2.3). Put together, the MaTeGnu approach, which systematically addresses BMMs as deep structure feature of teaching throughout the TPDP, in the events, the field-tested teaching materials, and the coaching and reflection in the PLCs (conceptual offer and support, see Chapter 5.2), appears to facilitate the transfer process.

### 9.2 Utilization of basic mental models in MaTeGnu classes compared to others

As outlined in Chapter 7.3 we assume the data of BMMD (Likert-Scale) as interval scale in accordance with the authors of the underlying GV-A test instrument to compare the findings with their evaluation study (Greefrath et al., 2023). This idealization is widely used in educational research context but not undisputed (Wu and Leung, 2017). As for our study, the idealization together with the even number of response options, which is required for our dichotomous analysis to answer RQ1, and missings resulting from the multi-matrix-design might explain the mediocre reliability values of the subscales of the BMMD in the experimental group (Kusmaryono et al., 2022).

The largest difference between experimental and control group with an almost medium effect shows the utilization ratings for the basic mental model rate of change (RC), followed by local linearity (LL) with a small effect. The ratings of tangent slope (TS) and amplification factor (AF) are also higher in the experimental group, but the difference is not significant, which might be explained by the general high (in case of TS) respectively low (in case of AF) level of ratings. There is also a slight non-significant decrease of agreement for formal-symbolic (FS) explanations in the experimental group.

The low level of AF ratings is in line with previous research, reporting students’ difficulties with and reluctance of the use of this basic mental model (Mamolo and Zazkis, 2012). The high ratings for TS are in line with the observations from research on classroom discourse, where teachers commonly illustrate the concept of derivative with the transition from secant to tangent line (Park, 2015). Above these levels our results clearly demonstrate a considerable impact of the MaTeGnu TPDP on students’ conceptual understanding.

On the level of individual tasks, ratings for the basic mental models RC, TS, LL are higher and ratings for FS are lower in both groups in contextualized tasks (2, 3, 4 and 7) than in other tasks, which empirically underpins the role of basic mental models as facilitator for the connection between mathematics and real-life situation (see Chapter 2.3).

A peculiar result is the low rating for task 6, where students are presented with the inner-mathematical problem to explain why the derivative function is doubled when the function itself is doubled. The tangent slope with the ideas of secant and gradient triangle or the amplification factor seem useful here from a theoretical perspective, but the data shows the opposite in both groups. Although the formal-symbolic explanation seems close to the algebraic expressions in the prompt of the task, the ratings in both groups are low. The students seem to see no benefit of formal expression when explaining this formal situation. The high ratings for rate of change might be due to the potential of this basic mental model to connect a context to the algebraic representation (Crawford and Scott, 2000). Following this argumentation the higher ratings in this task in the experimental group might indicate, that the MaTeGnu students seem to be more capable to exploit this benefit of rate of change. Local linearity has only slightly lower scores, which might be attributable to the focus on linear functions in lower secondary school mathematics.

Regarding RQ2a we can conclude that students in MaTeGnu mathematics classes show significant higher utilization of basic mental models, especially rate of change and local linearity than in other mathematics classes. The utilization of tangent slope is high for all students, except for the task addressing the algebraic representation (task 6), where all students utilize rate of change and local linearity with a slight lead for the MaTeGnu students. Formal-symbolic explanations show relatively low utilization, in the group of MaTeGnu students even lower.

The overall high level of utilization of tangent slope and the striking ratings in task 6 together with the high correlation between tangent slope and local linearity (as presented in the previous chapter) provides further empirical support for the teaching concept for derivatives as outlined in Chapter 5.2.3, i.e., to set a strong focus on rate of change for conceptualization of derivatives (as proposed by Crawford and Scott, 2000) and then relate to the graphical representation with tangent slope.

The significantly higher utilization of the basic mental models rate of change, tangent slope and local linearity by MaTeGnu students is remarkable empirical evidence of the success of the MaTeGnu TPDP, particularly the accompanying transfer, according to our TPDP transfer process model (see Figure 3), since the results are located on the most distal level of impact. The MaTeGnu concept, in particular with its conceptual and organizational support (cooperation and coaching at PLCs, aligned content, core practices; see Chapter 5.1 and 5.2 for details) enables the teachers to develop their teaching towards fostering their students’ development of basic mental models of derivatives and their utilization of basic mental models.

### 9.3 Conceptual understanding in MaTeGnu classes compared to others

The mediocre level of reliability of the CUD test as total scale can be partly explained by the planned missingness of the multi-matrix-design of the study. The good reliability measure of the CUD in the Rasch-model (see Chapter 8.1), which is more robust to missings, supports this argument and justifies the use of the test.

In the CUD MaTeGnu students demonstrate a notably higher level of conceptual understanding than other students. They score most different from other students in the contextualized tasks, a significant and almost medium effect. In the formal-symbolic tasks with graphs (GF) MaTeGnu students show higher results (EG: *M* = 2.07; CG: *M* = 1.66) than students in other mathematics classes as well, but the significance level and effect size reported in Chapter 7.3 can only be regarded as indications due to the reliability of the subscale. MaTeGnu students achieve slightly lower scores in the tasks of graphical derivation (GD) than other students. These tasks mainly address tangent slope, which is dominant in the control group. In addition, students often rely on trained routines when working with function graphs on derivatives (Asiala et al., 1997). Accordingly, the students in the control group could be somewhat more successful in these tasks by using solution routines.

Compared to Klinger’s (2018) validation study, our results reveal higher solution rates, particularly in the experimental group and difficult tasks show the largest differences. This suggests that the MaTeGnu teaching approach, which emphasizes understanding, provides a broader range of students with access to solutions for more difficult tasks.

Remarkably the solution rates of task Z8PC from the formal-symbolic subscale (GF) roughly double in the MaTeGnu group. In this task with a focus on TS students must decide upon properties of the derivative function based on the graph of the function. In contrast to the GD tasks, they cannot use routines here but must apply TS productively. MaTeGnu’s teaching concept seems particularly effective here. It supports the connection between the derivative concept and algebraic expressions by introducing TS as graphical interpretation relatively late, after the derivative concept has been established as the local rate of change.

Regarding RQ2b we can conclude that students in MaTeGnu mathematics classes show significant higher conceptual understanding than students in other mathematics classes. MaTeGnu students particularly use their understanding productively in contextualized tasks. Regarding the MaTeGnu TPDP concept, the results at the most distant level of TPDP impact show that focusing specifically on the transfer process (TPDP transfer process model, see Figure 3) is effective. This finding underscores the efficacy of the conceptual and organizational support framework within MaTeGnu, particularly evident in the initial implementation of the adapted teaching concept by participating teachers within the MaTeGnu mathematics classes.

### 9.4 Limitations of the study

As alluded in the discussion above, one of the empirical limitations is the reliability of the CUD and the simplification of the BMMD as interval scale in the analysis, but the good reliability scores of both tests in the IRT analysis demonstrate the suitability of the test instruments for this study.

Another restriction of the empirical data is the unbalanced sample of experimental and control group, which is due to joint data collection for all MaTeGnu topics of the control group (derivatives, integrals, analytical geometry and matrices, statistics). The test time was predetermined and the teachers of the participating mathematics classes decided upon the topic of their test depending on the previous teaching topic. This led to a considerable overlap of test data for the topic derivatives.

The ratings of utilization of basic mental models are only a predictor of the actual use of basic mental models in problem solving and the students’ own conceptual reasoning. An accompanying validation study, in the form of cued retrospective think-aloud interviews on the contextualized CUD tasks, is planned for the next evaluation cycle of MaTeGnu in the topic of derivatives.

The experimental group of this study is drawn from the MaTeGnu teacher educator qualification cycle. The teachers who participate in this cycle are experienced teacher educators (as outlined in Chapter 5.1.2) and hence incorporate high qualifications and deepened insights into contemporary teaching. This might lead to a higher level of teaching quality before the start of the TPDP and through dissemination at their schools to higher CUD and BMMD scores in the control group (drawn from mathematics classes of the schools of the teacher educators right before the start of MaTeGnu) and thus lower effects visible. On the other hand, the MaTeGnu teacher educators probably incorporate a special motivation to transfer the MaTeGnu teaching concept into their own teaching to gain experience for their work as MaTeGnu teacher educators, especially in the PLCs (see Chapter 5.1.4) and for their contribution to the maturation of the MaTeGnu teaching material in the expert team (see Chapter 5.2.2). Overall, we assume that the two effects roughly balance each other out and that this study is a good proxy for the evaluation cycle with the first cohort of teachers, who just started in summer 2025.

The TPDP is implemented as a state-wide professional development program, tailored to the state mathematics curriculum and the data is collected in schools throughout the state. However, the nationwide graduation exams and educational standards ensure the applicability of MaTeGnu in other states of Germany. On international level it might be necessary to realign the content to the local curriculum to ensure relevance of content and tailored support for implementation. The TPDP transfer process model presented in this article provides the criteria to adjust the specific MaTeGnu content and outline to the local prerequisites. However, we would recommend engaging all relevant institutions in the program as outlined for MaTeGnu in Chapter 5.1 to foster teaching development systematically.

## 10 Conclusion

MaTeGnu, a professional teacher development program for technology-enhanced teaching, aims to address two significant deficiencies in mathematics education at the upper secondary level:

1. The considerable number of students who fail to attain even a basic level of mathematical competence by the end of upper secondary education,
2. The low and even declining digital proficiency of German students, and the low proportion of teachers who incorporate digital technologies into mathematics classes.

MaTeGnu’s distinctive approach to addressing these issues involves the integration of digital technology to facilitate conceptual understanding through emphasizing basic mental models. The TPDP incorporates a meticulously structured concept that spotlights the transfer into teaching (TPDP transfer process model, Figure 3) and involves key stakeholders of the educational environment (see Chapter 5.1). The presented evaluation study offers results at the most distant level of TPDP impact, that is the students’ learning, which corroborate this transfer centered approach.

Students in MaTeGnu mathematics classes demonstrate significantly higher utilization of basic mental models together with significantly higher conceptual understanding. Consequently, it can be concluded that the MaTeGnu approach of TET with BMM promotes teaching with technology that fosters conceptual understanding. An in-depth analysis of the tasks reveals that the BMM local rate of change appears to facilitate the effective utilization of the BMM tangent slope. This finding lends empirical support to the particular design of the digital learning environments, in which derivatives are conceptualized as a local rate of change and subsequently represented graphically as tangent slope. In light of the findings, it can be recommended to employ this design for the teaching of derivatives, even on an international scale. Furthermore, it is advised that a corresponding remark be incorporated into curricula. The specific teaching material^4^ has been developed based on the regional mathematics curriculum and is fully compliant with the German educational standards in mathematics, i.e., it is applicable throughout Germany. In order to ensure optimal functionality on an international scale, a comprehensive review of the compatibility of individual components with national curricula is imperative. This evaluation process enables the selection of components that are compatible with the specific educational standards and objectives of each nation.

The evaluation concept of MaTeGnu incorporates empirical studies on each of the five topic modules (derivatives, integrals, analytical geometry and matrices, statistics, and exponential functions). The preliminary data analysis on conceptual understanding and utilization of basic mental models on integrals shows similar results. Consequently, our data indicates that the prioritization of BMM may offer an effective strategy to leverage technology’s benefits for learning and to overcome teachers’ perceptions mindless working when teaching with technology (Thurm and Barzel, 2022). This approach has the potential for application even in the more formal setting of upper secondary school mathematics.

The distinct results on the most distant level of TPDP impact also underpin MaTeGnu’s effort to support the participating teachers in their transfer process from TPDP to actual teaching (TPDP transfer process model, see Figure 3). Given the assumption that focusing on understanding using basic mental models (deep structure) necessitates a substantial realignment of rather formal teaching directed towards academic aptitude, MaTeGnu particularly addresses the transfer process with conceptual and organizational support.

This support of the transfer process also incorporates the paradigm of constructive alignment of learning and assessment (Biggs, 1996) in the PLCs. This process encompasses an additional dimension of MaTeGnu’s evaluation framework, situated at impact level three: the teaching practice. The respective study compares the assessments MaTeGnu teachers pose before the start of TPDP and in the TPDP regarding the addressed basic mental models and conceptual understanding. Preliminary analyses indicate an increase in both aspects for assessments on derivatives.

In summary, the tangible concept for the use of technology to promote understanding, implemented in evidence-based teaching material, in conjunction with the facilitation of the transfer process into the teacher’s pedagogical practice appears to be the pivotal combination for the efficacy of MaTeGnu.

## Data Availability

The raw data supporting the conclusions of this article will be made available by the authors without undue reservation.

## Footnotes

^1^ https://o-mathe.de

^2^ https://m2.net-schulbuch.de

^3^ https://www.r-project.org

^4^ https://mategnu.de/m/l1e
